# Plant–microbe interactions in the apoplast: Communication at the plant cell wall

**DOI:** 10.1093/plcell/koac040

**Published:** 2022-02-14

**Authors:** Susanne Dora, Oliver M Terrett, Clara Sánchez-Rodríguez

**Affiliations:** Department of Biology, ETH Zurich, 8092 Zurich, Switzerland; Department of Biology, ETH Zurich, 8092 Zurich, Switzerland; Department of Biology, ETH Zurich, 8092 Zurich, Switzerland

## Abstract

The apoplast is a continuous plant compartment that connects cells between tissues and organs and is one of the first sites of interaction between plants and microbes. The plant cell wall occupies most of the apoplast and is composed of polysaccharides and associated proteins and ions. This dynamic part of the cell constitutes an essential physical barrier and a source of nutrients for the microbe. At the same time, the plant cell wall serves important functions in the interkingdom detection, recognition, and response to other organisms. Thus, both plant and microbe modify the plant cell wall and its environment in versatile ways to benefit from the interaction. We discuss here crucial processes occurring at the plant cell wall during the contact and communication between microbe and plant. Finally, we argue that these local and dynamic changes need to be considered to fully understand plant–microbe interactions.

## Teaser

Plants and microbes modify the host cell wall to benefit from the interaction by altering its properties, which are defined by the biochemistry of its polysaccharides, regulated by cell wall ions and proteins.

## Introduction

**Figure 1:**
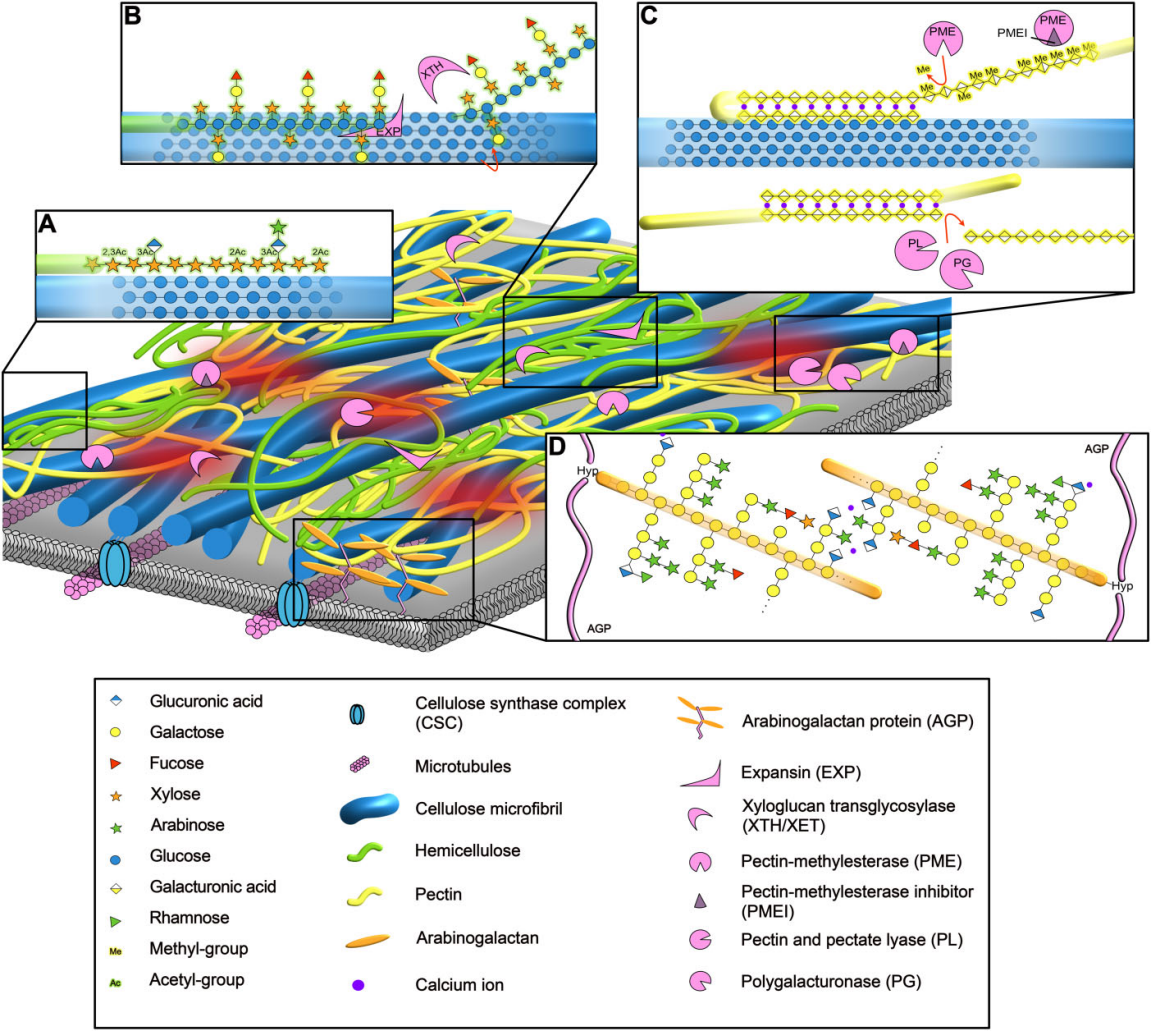
A structural model of the primary CW. Based on atomic force microscopic images of onion epidermal CWs (Cosgrove, 2014): cellulose microfibrils (blue fibrils) are shown embedded in a matrix of pectins (yellow chains), hemicellulose (green chains), arabinogalactan proteins (linear protein backbones are shown in pink with glycosylated hydroxyproline residues in orange), and other plant CW proteins (pink). Limited areas, called biomechanical hotspots (red shadows), are thought to contribute to CW mechanics disproportionately, and likely include sites of contact between cellulose and other molecules. The cellulose microfibrils are synthesized at the plasma membrane by cellulose synthase complexes tracking along microtubules, while the other polysaccharides are synthesized in the Golgi and assembled at the apoplast. A, Xylan is shown binding to the hydrophilic face of cellulose microfibrils via hydrogen bonding, in the same conformation as the glucan chains within cellulose. B, Xyloglucan binds to the hydrophobic face of cellulose based on molecular dynamics simulations, though details of this interaction require further investigation. This interaction is modified by EXP and XTH/XETs. EXPs are nonhydrolytic proteins that cause CW loosening through an unknown mechanism, most probably by separating hemicelluloses and cellulose that are interacting. An XTH has transglycosylated a xyloglucan chain onto a glucan chain of cellulose, forming a new covalent bond, highlighted in red. XTHs can also make xyloglucan–xyloglucan links, or hydrolyse xyloglucan, also (data not shown). C, HG is demethylated by PMEs that are inhibited by PMEIs, regulating the methylation status of the pectin. PME-demethylated regions of HG bind Ca^2+^ (fuchsia circles), forming dimerized egg-box structures, either intra- or intermolecularly. Ca^2+^-bound pectin seems to associate with cellulose, though the details of this interaction are not well understood. The size of demethylated HG is reduced by the cleavage of pectate and PLs and PGs. D, The glucuronic acid residues of arabinogalactan proteins can bind Ca^2+^, and this may enable dimerization.

The apoplast is the compartment of the plant between the plasma membrane and the external surface (Sattelmacher, 2001). This space contains the extracellular domains of plasma membrane proteins; cell wall (CW) polysaccharides, proteins, polyphenols, and ions; and water and air. The apoplast has essential roles in plant biology including photosynthesis, transpiration, water and nutrient uptake, and movement of signaling molecules (Figure 1). During plant colonization by all nonviral microbes, the apoplast is the space where the interaction is initially established and where the intruder lives for most, if not all, of its lifetime inside the host. The apoplast is, therefore, a site of intense activity as the interface for plant–microbe communication.

**Figure 2:**
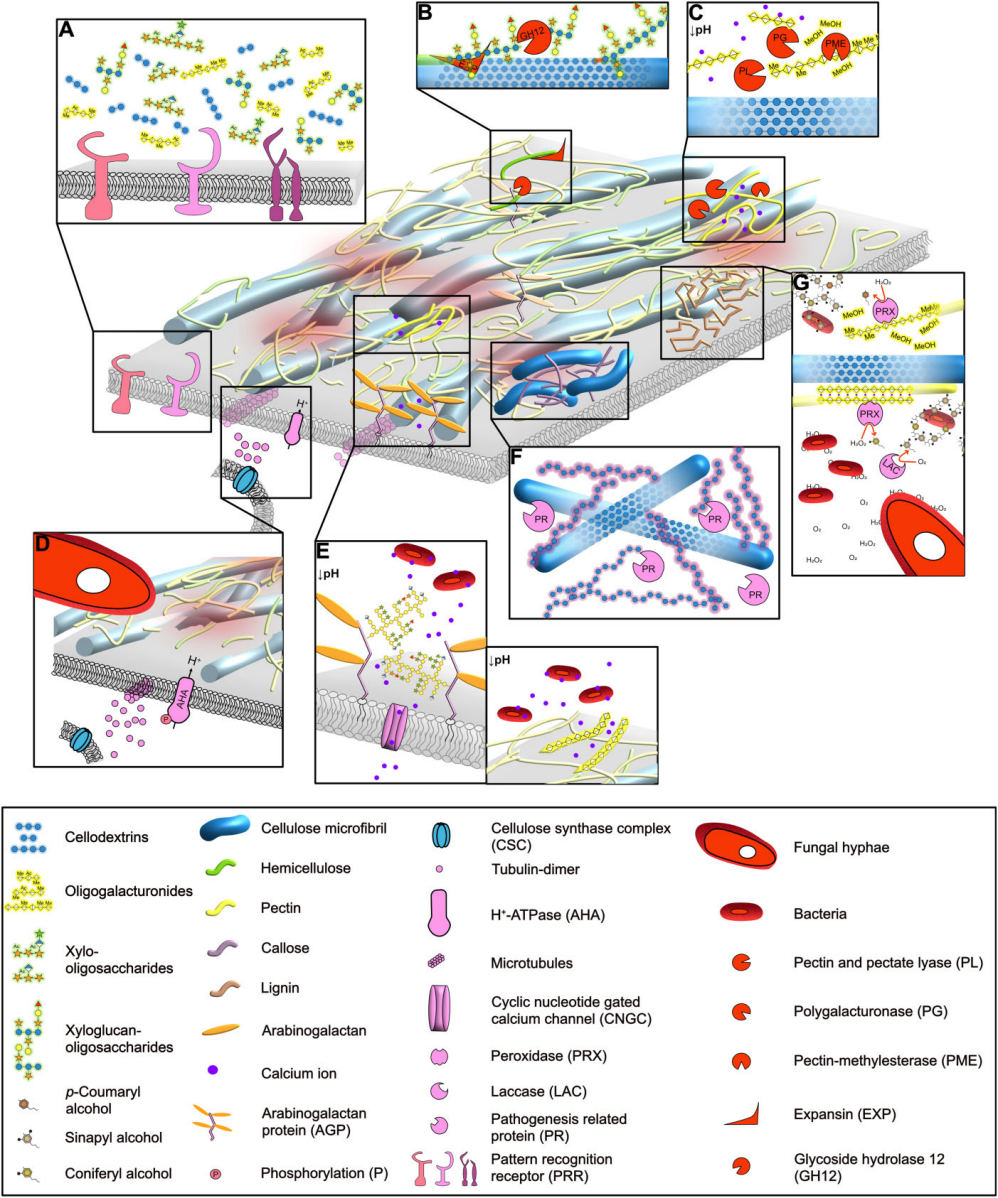
Modification of the plant CW environment upon microbial colonization. The polysaccharides of the primary CW represented in Figure 1 have been loosened and degraded by microbial CWDE and CW-modifying enzymes (red), and the CW ionic environment has been modified (A–E). The plant responds by synthesizing new CW material (F–G). For elements present in Figure 1, refer to its key. A, Plant CW polysaccharides become digested upon microbial enzymatic activities. Some of the resulting oligosaccharide fragments are detected as DAMPs by plasma membrane localized receptors that induce signaling cascades leading to defense reactions. Shown are confirmed DAMPs, such as cellobiose and OGs, and potential DAMPs, such as xyloglucan- and xylan-polysaccharides. B, Microbial xyloglucanases (GH12) and EXP cut the xyloglucan and loosen the **Figure 2** (Continued) xyloglucan–cellulose interactions, respectively. This may reduce cross-linking between microfibrils enabling separation/sliding of the cellulose microfibrils, which could be important for CW loosening. Other hemicelluloses are targeted by CWDEs from different CaZY families, not shown. C, Microbial PMEs remove methyl groups from HG, while pectate and PLs and PGs cut this polymer into smaller fragments. At low pH, or potentially small HG size, the HG loses the ability to bind Ca^2+^ ions, possibly reducing the interaction between HG and cellulose. D, Apoplast acidification through hyperactivation of the plant plasma membrane proton ATPases (AHAs) in response to *F. oxysporum* contact, leads to the depletion of cellulose synthase complexes from the plasma membrane and cortical microtubule depolymerization. This leads to a halt in cellulose synthesis and, may be a general response to pathogen infection, though has, thus far, only been observed in *F. oxysporum*. E, A drop in apoplastic pH leads to a release of Ca^2+^ ions from arabinogalactan proteins and HG, which could contribute importantly to the cytosolic Ca^2+^ peak detected in response to microbes. The liberated Ca^2+^ can be sequestered by bacterial extracellular polysaccharides, affecting the Ca^2+^ dynamics that are important for activating plant immunity. F, In response to some microbial attacks, the plant cell forms a papillae composed of cellulose, callose (purple chains), PRs, and secondary metabolites with antimicrobial properties. The papillae polysaccharides interact in unknown ways, though calllose modifies the extensibility of cellulose gels in vitro. G, The plant cell reinforces its CW by lignification (light brown chains) through the activity of PRXs and LACs. PRXs binding to demethylated HG, which might help localize lignin formation at sites of CW damage. Some microbes get trapped in the newly formed lignin-network. Microbes can scavenge ROS through their extracellular polymeric substances. Many of the models shown are speculative, based on research in related contexts (C, D, F, and G) or the known activities of well-described enzymes (E and F).

The interaction between microbes and plants ranges from mutualism to parasitism. Pathogens are further categorized, based on their different lifestyles in the host, as biotrophs, necrotrophs, and hemibiotrophs, though there is some debate about the accuracy of these categories (Rajarammohan, 2021). Whatever the type of interaction, the microbe requires plant CW loosening and degradation in part for nutrition and in part to make space for growth (Kubicek et al., 2014). Some of these plant CW alterations caused by the intruder release CW fragments that are recognized by the host as damage-associated molecular patterns (DAMPs) through plasma membrane pattern recognition receptors (PRRs), which bind the released CW fragments (DeFalco and Zipfel, 2021; Figure 2A). Other PRRs recognize microbe-associated molecular patterns (MAMPs) like chitin oligomers released from fungal CWs by the action of plant apoplastic enzymes. MAMP and DAMP recognition by PRRs triggers plant pattern-triggered immunity (PTI) that results in important apoplast alterations acting as basal defenses, including apoplastic pH changes, Ca^2+^ release from the CW and influx to the cytosol, and production of reactive oxygen species (ROS). The signal transduction and activation of transcriptional responses to the intruder, amplified by various phytohormonal signals, leads to a complex defense reaction. The plant’s response is specific to each infection, but includes programmed cell death, the secretion of antimicrobial peptides, pathogenesis-related (PR) proteins, and proteases into the apoplast, and structural modifications of the CW. The microbe must circumvent these apoplastic defenses to survive in the plant. To this end, microbes have evolved different mechanisms to minimize their detection by the host and to block the MAMP/DAMP-triggered defense.

For decades the study of plant responses to molecular pattern perception has received most of the attention in the field of plant resilience to biotic stress. In light of emerging methodologies, including high-resolution microscopy, structure-resolving techniques like solid-state nuclear magnetic resonance (NMR), and complex computational simulations, the architecture and mechanics of the plant CW are regaining their leading role in this area. Thus, in this review, we will avoid discussion of the details of DAMP/MAMP perception, the ensuing intracellular signaling pathways, and some downstream responses, which are well-reviewed elsewhere (Coaker, 2020; Zhou and Zhang, 2020; Bizuneh, 2021; DeFalco and Zipfel, 2021). Instead, we will focus on the plant CW as an active element in the interaction, describing how it is modified by the microbe, and reinforced by the plant, and how this relates with ion movement and changes in extracellular protein activity. This review will largely focus on pathogenic microbes and will not include an exhaustive comparison between mutualists and pathogens.

## The plant CW

The plant CW is a heterogeneous mixture, mainly composed of polysaccharides (Burton et al., 2010). The properties of different CWs vary, even surrounding the same cell, due to interactions between CW components. To adapt to changing cellular and organ needs, CW properties can be modified by apoplastic proteins, changing ionic concentrations, and by deposition of new material. Here, we largely describe the composition of eudicot primary CWs, exemplified by the model plant *Arabidopsis thaliana* (Burton et al., 2010). However, the type and structure of polysaccharides, particularly hemicelluloses and pectins, varies phylogenetically, reviewed elsewhere previously (Scheller and Ulvskov, 2010). Within the same plant, CW composition and thickness varies in different tissues, such as the CWs of xylem vessels and tension wood, though this is outside the scope of this review (Burton et al., 2010; Gorshkova et al., 2015).

CW polysaccharides include cellulose, hemicelluloses, and pectins. The cellulose is synthesized at the plasma membrane by cellulose synthase complexes tracking over cortical microtubules (Figure 1), while pectins and hemicelluloses are synthesized in the Golgi (Paredez et al., 2006; Mohnen, 2008; Scheller and Ulvskov, 2010). During cellulose synthesis, the β-1,4-glucan chains assemble into semi-crystalline microfibrils that form the structural base of the CW, contributing to its strength, tissue structure, and function (Somerville, 2006). Cellulose has been reported to bind hemicelluloses, facilitated by their structurally similar β-1,4-glycosidic bonds (Figure 1, A and B; Dick-Pérez et al., 2011; Simmons et al., 2016; Kang et al., 2019; Terrett et al., 2019). The composition of hemicelluloses is complex and varies in different phylogenetic groups, but hemicelluloses all share backbone β-1,4-glycosidic bonds, though some hemicelluloses have additional backbone glycosidic bonds (Scheller and Ulvskov, 2010). These interactions are thought to influence wall strength, supported by the developmental defects shown by many hemicellulose mutants (Scheller and Ulvskov, 2010; Grantham et al., 2017). The binding of hemicelluloses to cellulose microfibrils may prevent cellulose aggregation and enables cross-linking of microfibrils to each other or to different CW components, like the phenolic polymer lignin (Kang et al., 2019; Terrett et al., 2019). The prevention of cellulose aggregation could be important to maintain the orientation of microfibrils in the different lamellae of the wall, which may contribute to resistance to mechanical forces in different directions (Zhang et al., 2017). Any potential cross-linking between microfibrils or to other components, may prevent slippage of microfibrils, which is thought to contribute to cell expansion (Zhang et al., 2021c). The sites of interaction between cellulose and the hemicellulose xyloglucan, called “biomechanical hotspots” (Park and Cosgrove, 2012a), influence the biomechanical properties of the wall (Park and Cosgrove, 2012b) and are thought to be the target of expansins (Figure 1B), proteins which loosen the CW (Wang et al., 2013). The exact structure of the biomechanical hotspots is not fully understood. Endogenous xyloglucan transglycosylases (XTH/XETs, Figure 1B) also remodel xyloglucan and covalently link it to cellulose and other xyloglucans (Van Sandt et al., 2007; Wang et al., 2016). Similar remodeling activities have been identified for other hemicellulosic polysaccharides in some plants (Franková and Fry, 2021). The action of apoplastic CW-modifying proteins, like expansins and XTH/XETs, is regulated by pH (Sampedro and Cosgrove, 2005; Shi et al., 2015).

In contrast to the structurally similar cellulose and hemicelluloses, the other major polysaccharide component of plant CWs, especially in eudicots, are pectins. Pectins are mainly composed of homogalacturonan (HG), a backbone that can be decorated with different sugar branches, forming different pectic polysaccharides, including rhamnogalacturonan-II (Pellerin et al., 1996; Mohnen, 2008). HGs are secreted in an acetyl- and heavily methyl-esterified form. Once at the apoplast, they are demethylated by CW pectin methylesterases (PMEs) and deacetylated by pectin acetyl esterases (Pauly and Ramírez, 2018; Wu et al., 2018). PME activity is regulated by pectin-methylesterase inhibitors (PMEIs), among other actors, such as the proteases which can cleave the PME’s auto-inhibitory domains (Bosch et al., 2005; Wormit and Usadel, 2018). PME activity is heavily affected by pH, by altering the interaction of PMEIs and PMEs (Sénéchal et al., 2017; Pauly and Ramírez, 2018; Wu et al., 2018), and by changing the processivity of PMEs, which affects the formation of blocks of demethylated residues (Wormit and Usadel, 2018; Hocq et al., 2021). Demethylated regions of HG can bind Ca^2+^, producing the so-called “egg-box” structures that form gels (Figure 1C; Jarvis and Apperley, 1995; Zdunek et al., 2021). The egg boxes are thought to enable dimerization of different pectin chains, with effects on the mechanical properties of the CW (Zdunek et al., 2021). The Ca^2+^ binding is affected in vitro by acetylation of HG, which might also affect the conformation of the backbone (Renard et al., 1999). Although their backbone has a different shape to that of hemicelluloses and cellulose, HG can also interact with cellulose (Wang et al., 2015; Figure 1C). The extent of this interaction and the rigidity of pectin correlates positively with demethylation (Phyo et al., 2017a). Consistent with this, the egg box HG seems to be closer to cellulose in space (pre-print Temple et al., 2021). The binding of Ca^2+^, and thereby the conformation of HG, and its interaction with cellulose, is regulated by pH, as protonation of HG at low pHs stops the ionic interaction with Ca^2+^ (Phyo et al., 2019). On the other hand, HG demethylation may loosen the wall, allowing cell expansion. HG demethylation, observed in a band pattern in epidermal CWs (Haas et al., 2020), was predicted to allow expansion of those areas of the CW, leading to the formation of lobes in epidermal cells, though this model is controversial (Cosgrove and Anderson, 2020). The overexpression of polygalacturonases, enzymes that hydrolyse HG, some of which prefer demethylated HG as a substrate, also leads to increased cell expansion and decreased pectin–cellulose interactions, indicating that smaller pectins interact less with cellulose (Phyo et al., 2017b). Thus, pectin demethylation has been reported to both increase and decrease CW stiffness, depending on the experimental system used (Bou Daher et al., 2018; Altartouri et al., 2019; Wang et al., 2020a). In summary, while demethylation may enable cross-linking of HG chains and binding to cellulose, it also makes the HG more vulnerable to digestion by pectate lyases and hydrolases that prefer charged pectin, which may explain contradictory effects on CW mechanics. Some of these different effects may arise from differences in the pattern of demethylation that is produced, which may make pectin more or less likely to chelate calcium or be cut by lyases and hydrolases (Wormit and Usadel, 2018).

Some pectins and the hemicellulose xylan have been reported to be covalently linked to arabinogalactan proteins (AGPs), though it is unclear how widespread the interaction is (Tan et al., 2013). AGPs sit at the interface between polysaccharide and protein, with a largely unfolded protein backbone that is heavily glycosylated (Figure 1D). The glucuronic acid (GlcA) residues at the end of AGP side-chains can bind to Ca^2+^ (Tryfona et al., 2012; Lamport and Várnai, 2013). Most classical AGPs are attached to the plasma membrane through a glycosylphosphatidylinositol anchor, but some are released into the wall.

CWs also contain the nonpolysaccharide polymers lignin and suberin. Lignin, a hydrophobic phenylpropanoid polymer (Ralph et al., 2004; Tobimatsu and Schuetz, 2019), can cross-link to other CW components (Terrett and Dupree, 2019), including polysaccharides and potentially proteins (Diehl and Brown, 2014; Preis et al., 2018). Pectin monomers are biosynthesized in the cytosol and polymerized in the apoplast by laccase and peroxidase proteins (Tobimatsu and Schuetz, 2019). The polymerization of lignin undergoes tight spatiotemporal control, normally found in select tissues, such as the xylem vessel, fiber cells, and the Casparian band, it can also be produced in response to pathogens (Lee et al., 2013; Barbosa et al., 2019). Suberin is a hydrophobic lipid-based polymer that can form part of the CWs of infected or wounded cells (Philippe et al., 2020).

We now have a reasonably detailed description of some of the major interactions arising between CW polysaccharides (Figure 1). However, a cohesive model of those interactions and their relationship to the emergent properties of the CW, and especially of whole cells or tissues, is still being developed. This is particularly relevant to plant–microbe interactions, where we need to understand how CW properties and structure relate to the development of disease.

## Microbial modification of plant CW environment

### CW polysaccharides

Microbes need to modify the plant CW to properly interact with the host. With this aim, they utilize noncatalytic proteins with important CW loosening effects, such as expansins and expansin-like proteins, loosenins, and swollenins, which were recently reviewed (Georgelis et al., 2015; Narváez-Barragán et al., 2020). Together with these proteins, intruders secrete an array of CW degrading enzymes (CWDEs) classified into families in the carbohydrate active enzyme (CaZY) database, based on their catalytic mechanism and sequence similarity: glycosyl hydrolase (GH), polysaccharide lyase (PL), carbohydrates esterase (CE), and auxiliary activity (AA) including lytic polysaccharide monooxygenase (LPMO; Lombard et al., 2014). The CWDEs remove substitutions, disrupt the interactions between polysaccharides, and break them into smaller fragments (Kubicek et al., 2014), thereby affecting the CW structure, though these effects are poorly understood (models of some potential structural changes are shown in Figure 2). The amount and variety of CWDEs secreted by pathogens correlates somewhat with their lifestyle and host (King et al., 2011; Zhao et al., 2013; Hane et al., 2020). In general, necrotrophs have a greater number of CaZY families in their genome than biotrophs (Kubicek et al., 2014). This is largely because necrotrophs kill host cells rather than using them as a continuous supply of primary metabolites, thus they must degrade and feed on the complex polymers of the CW (Hane et al., 2020). In contrast, many biotrophs and mutualistic microbes have a reduced number of CWDEs and may rely instead on endogenous plant CWDEs (Balestrini and Bonfante, 2014; Rich et al., 2014).

Pathogen CWDE mutants provide strong evidence that the degradation of specific polysaccharides is important for the development of disease. Some pathogens with reduced cellulase arsenals are impaired in virulence. For example, the absence of a single cellulase in the bacteria *Dickeya dadantii* and *Clavibacter michiganensis* decreased the severity of visible disease symptoms (Mäe et al., 1995; HWang et al., 2019). Knockdown of nine predicted cellulases, members of the GH6 and GH7 families, also reduced *Magnaporthe oryzae* virulence, potentially due to an increase in papillae formation or the diminished papillae-degradation capacity of the fungus (Van Vu et al., 2012). However, recent data show that *Fusarium oxysporum* with low cellulose-degradation capacity advance faster through the apoplast and are hypervirulent, but are impaired in saprophytic growth and reproduction (pre-print Gámez-Arjona et al., 2021). Thus, cellulose is an important structural barrier to the establishment of disease for some pathogens, while for others its degradation is mainly important for the saprophytic phase.

Hemicellulose and pectin degradation is also relevant for pathogenesis. Xyloglucan degradation seems to be important for the infection of some filamentous pathogens, as reported for *Phytophthora sojae* and *F. oxysporum* mutants, which were considerably less virulent when lacking a single GH12 xyloglucanase (Figure 2B; Ma et al., 2015; Zhang et al., 2021b). In contrast, the hydrolytic activity of a *Botrytis cinerea* xyloglucanase is unimportant for its function in planta, though the enzyme induces cell death regardless of its activity (Zhu et al., 2017). Similarly, two *Verticillium dahliae* GH12 xyloglucanases also cause cell death in cotton (*Gossypium hirsutum*), reducing the virulence of the fungus, in the absence of these enzymes, the fungus becomes more virulent (Gui et al., 2017).

Other hemicelluloses are important for disease resistance and there are numerous reports of the importance of pathogen xylanases in the development of disease in Angiosperms. Xylanase mutants in the fungi *V. dahliae* (Wang et al., 2021a) and *M. oryzae* (Wu et al., 2016), the bacteria *Xanthomonas oryzae* (Rajeshwari et al., 2005), and the oomycete *Phytophthora parasitica* (Lai and Liou, 2018) have reductions in virulence. Xylanase activity is not always essential though, even for infection of plants with high xylan content, such as wheat, in *Fusarium graminearum* infection of wheat and soybean, deletion of a regulator of xylanase expression had little effect on symptoms, despite a drop in xylanase activity (Paccanaro et al., 2017). Demethylation of pectin by *F. graminearum* PMEs increases the percentage of symptomatic infected wheat spikelets (Sella et al., 2016). Mutations in a pectate lyase in *V. dahliae* (Yang et al., 2018), a polygalacturonase in *R. solanacearum* (among other examples; Huang and Allen, 1997, 2000; Basaran et al., 2007; Thilini Chethana et al., 2020), and in the newly discovered HG-specific LPMO in *Phytophthora infestan*s compromise virulence (Figure 2B; Sabbadin et al., 2021). On the other hand, a *F. graminearum* polygalacturonase mutant has similar virulence in wheat and soybean to the wild-type fungus (Paccanaro et al., 2017). It is clear that some plant CW degradation is important for pathogenesis and mutualism, and most species require degradation of multiple polysaccharides, but the importance of degrading a particular CW component varies in different microbial species, exemplified by examples where CWDE mutants enhance or have no effect on virulence. The importance of CW degradation can be concealed by the degradation of other CW components; for instance, the importance of xylan and pectin degradation for *F. graminearum* is only apparent in a double mutant, which has lost both activities (Paccanaro et al., 2017). The function of various polysaccharides in plant defense are also understudied from the perspective of microbial CWDE mutants, especially that of mannans and arabinogalactans (Villa-RiVera et al., 2021). In some cases, the main difficulty is the challenge of working with certain plants, such as gymnosperms, which have a high mannan content (Scheller and Ulvskov, 2010).

Pathogens require CWDEs for effective establishment of disease; however, the products of the CWDE activity can be identified as DAMPs by the plant, activating immune responses. Oligogalacturonides (OGs) from HG; cello-oligosaccharides from cellulose, mixed-linkage glucan, and xyloglucan; xylo-oligosaccharides from xylan; gluco-oligosaccharides from β-1,3glucans similar to callose, as well as xyloglucan-oligosaccharides, have all been reported to activate PTI (Nothnagel et al., 1983; Souza et al., 2017; Claverie et al., 2018; Mélida et al., 2018, 2020; Yang et al., 2021). Oligosaccharides from different polymers induce different immune responses; xyloglucan-oligosaccharides and OGs both reduce disease severity from *B. cinerea* and *Hyaloperonospora arabidopsidis* infection, but only the OGs induced a ROS burst when applied to leaves (Claverie et al., 2018). Cello-oligosaccharides also did not induce a ROS burst in Arabidopsis, but did upregulate defense-gene transcription, e.g. WRKY30 (Souza et al., 2017).

Plants can decode oligosaccharides with exquisite precision. For instance, the addition of a galactose and fucose disaccharide to a seven-sugar xyloglucan-derived oligosaccharide enables it to inhibit auxin-induced elongation of etiolated pea (*Pisum sativum*) stems (York et al., 1984). These different oligosaccharides, derived from the same polymer, are potentially the products of different CaZYmes. For example, GH30 xylanases produce longer xylo-oligosaccharides than GH11 xylanases, and the glucuronic acid substitution is found in a different position on these two xylo-oligosaccharides (Vrsanská et al., 2007). Plants and pathogens have differences in the CaZYme families in their genomes; for instance many fungi secrete GH12 xyloglucanases (Figure 2B) and many bacteria encode GH30 xylanases, neither of these GH families are present in the poplar (*Populus trichocarpa*) and Arabidopsis genomes (Kumar et al., 2019). Thus, plants might distinguish the CW degradation products derived from its own GHs during growth from those released by GHs from an intruder.

DAMP perception by PRRs is one of the most active areas of research in plant immunity and pathology and the topic of frequent reviews (Saijo et al., 2018; Hou et al., 2019; Lu and Tsuda, 2021). However, for most DAMPs, their PRR is unknown, and identifying such proteins will continue to be an important area of research, as we persevere in determining how specific but similar oligosaccharide structures generate different plant responses. This area is particularly complex, as PRRs are required to detect very specific structures, but also highly unrelated structures. For instance, the receptor CERK1, is involved in detecting short mixed linkage glucan (MLG) oligosaccharides and β-1,3-gluco-oligosaccharides, but not long β-1,3-gluco-oligosaccharides (Wanke et al., 2020; Yang et al., 2021). Despite this specificity in distinguishing different size similar structures, the *cerk1* mutant is affected in the detection of a variety of MAMPs, including chitin, lipopolysaccharides (LPS), and peptidoglycan (Desaki et al., 2018). Unexpectedly, CERK1 has been recently shown to be essential for the detection of mixed MLGs in Arabidopsis, rice (*Oryza sativa*) and barley (*Hordeum vulgare*), though this is controversial in Arabidopsis as different groups have shown opposite results in technically similar experiments (Barghahn et al., 2021; Rebaque et al., 2021; Yang et al. 2021). Interestingly, only monocots, not eudicots, contain MLGs in their CWs (Little et al., 2018). A structurally similar MLG has been found in oomycete CWs, and thus the capacity to detect MLG-oligosaccharides may be important for eudicots to detect MAMPs from oomycetes rather than DAMPs from their own CWs (Rebaque et al., 2021).

To reduce DAMP-induced PTI, pathogens attempt to control the plant perception of polysaccharide DAMPs. With this aim, some microbes secrete proteins that sequester the DAMPs, blocking their binding to the corresponding PRRs, e.g. the *Ralstonia solanacearum* lectin that binds fucose containing xyloglucans (Kostlánová et al., 2005). Other pathogens have evolved CWDEs whose degradation products are not yet recognized as DAMPs by the plant, like the pectin LPMOs from oomycetes (Sabbadin et al., 2021). LPMOs are known for their contribution to lignocellulose deconstruction, based on their ability to degrade crystalline and recalcitrant polysaccharides. Thus, the oomycetes might have evolved a novel LPMO activity whose products are predicted to attenuate PTI, which could mimic the plant mechanism to mitigate its PTI, by producing oxidized OGs (Benedetti et al., 2018; Pontiggia et al., 2020). The novel role of pectin LPMOs in reducing defense activation is a speculative idea which will need to be investigated further.

In contrast to this idea of the immunity-evading function of oxidative polysaccharide degradation, the products of a cellulose-specific LPMO caused immune responses in Arabidopsis, some of which were stronger than to the widely accepted DAMP cellobiose (Zarattini et al., 2021). In addition, some pathogens express AA7 enzymes, which are thought to oxidize oligosaccharides, and may be important for reducing the immunogenic potential of pathogen generated DAMPs (Pontiggia et al., 2020; Haddad Momeni et al., 2021; Wang et al., 2021b). Interestingly, plants utilize AA7 enzymes to reduce the immune-activating capacity of DAMPs (Benedetti et al., 2018; Pontiggia et al., 2020). Future research on the homeostasis of DAMPs, by plant and pathogen, will continue to shape the field in the coming years. The negative effects of DAMP production can outweigh the advantages of structural disruption of the CW. Thus, the elimination of certain microbial CWDEs can increase their pathogenicity, presumably as a side-effect of reduced DAMP production. For instance, two *M. oryzae* mixed-linkage glucanase mutants have increased pathogenicity and, accordingly, the DAMPs produced by active versions of these proteins cause MAP kinase activation in the host (Yang et al., 2021).

Recently, it has been demonstrated that CWDEs can be MAMPs. Some catalytically inactive CWDE mutant proteins induce PTI, indicating that the enzymes themselves, and not just the DAMPs they generate, are recognized by the plant (Ma et al., 2015). Three Brassicaceae species have been shown to recognize three different short peptides derived from fungal, and potentially oomycete, polygalacturonases (Zhang et al., 2021a). Many other plant species did not recognize these polygalacturonases as MAMPs, suggesting that CWDE-derived MAMPs are highly specific to a few interactions. These peptides are buried in the hydrophobic core of the polygalacturonases, suggesting the presence of plant proteases that digest the CWDEs and release these new MAMPs for recognition. Although the importance of extracellular proteases in plant immunity is well established (Wang et al., 2020b), their potential functions in releasing MAMPs from pathogen CWDEs remain to be fully investigated.

Microbial manipulation of the plant CW generates not only chemical but also mechanical signals (Bacete and Hamann, 2020). While CW loosening and cell separation are necessary to facilitate infection and make space for the growth of microbial cells or feeding structures (e.g. haustoria and arbuscules), the plant cell can perceive changes in the mechanical forces experienced by the plasma membrane and CW, as recently reviewed elsewhere (Codjoe et al., 2022). The mechanosensing can be important for generating an immune response, as mutants in some mechanosensitive proteins, such as the mechanosensitive channel of small conductance-like (MSL) plasma membrane channels, are affected in some downstream CW reinforcement responses and phytohormonal levels (Engelsdorf et al., 2018). The complex detection system evolved by plants integrates all these signals, activating an immune response that includes reinforcement of the CW, which will be discussed later.

Together with microbial mutants in CWDE and CW loosening proteins, plants impaired in the biosynthesis of certain CW components are used to understand CW-based disease resistance, recently reviewed elsewhere (Bacete et al., 2018). This approach has yielded important insights, especially into the interplay between CW structure, CW integrity monitoring, and phytohormone crosstalk. For instance, cellulose and pectin synthesis defects affect jasmonic, ethylene, and salicylic acid responses (among others), while hemicellulose synthesis defects can affect brassinosteroid and strigolactone responses (Bacete et al., 2018; Ramírez and Pauly, 2019; Menna et al., 2021). The responses can be highly specific even in mutants with defects in the same CW component. For example, different lignin synthesis mutants have little overlap in specific defense genes that are activated (Ha et al., 2021). Together with the study of plant CW mutants, the in planta expression of microbial CWDEs has revealed that structural changes of polysaccharides mediated by these enzymes, or the enzymes themselves are perceived by the plant (Swaminathan et al., 2021). For instance, the overexpression of fungal acetylesterases targeting pectin or xylan generates increases in defense gene expression. CW synthesis mutants with defects in pectin or xylan acetylation have resistance phenotypes, suggesting the importance of this polysaccharide structure (Manabe et al., 2011; Escudero et al., 2017). Despite these interesting discoveries, the interpretation of the structural roles of polysaccharides in the CW is complicated by the diverse hormonal and metabolic changes derived from CW modifications in these mutants (Hernández-Blanco et al., 2007; Menna et al., 2021). In addition, alterations in a certain CW component also affect the structure of the entire CW matrix (Huang et al., 2017; Park and Cosgrove, 2012a). Thus, understanding the whole CW structure is essential to assess the effect of a certain mutation.

### Apoplastic pH

Apoplast alkalization is reported as one of the first plant responses to MAMPs/DAMPs, together with a rise in cytosolic Ca^2+^ (Thor and Peiter, 2014; Moroz et al., 2017; Thor et al., 2020). The apoplast pH is mainly regulated by the activity of plasma membrane H^+^-ATPases (AHAs in Arabidopsis; Benschop et al., 2007; Nühse et al., 2007), channels that import H^+^ to co-transport other molecules, and anion efflux (Jeworutzki et al., 2010; Lehmann et al., 2021). The exact molecular mechanism of the origin and consequences of this reduction of H^+^ in the apoplast is still unknown but is required for plant defense against microbes, as shown in plants grown in acidic media (Yu et al., 2019). Therefore, to counteract the increase in pH, various microbes rely on apoplast acidification to colonize their host. Among them, the pathogenic fungi *Botrytis cinerea* and *Sclerotinia sclerotiorum* secrete citric acid and oxalic acid, respectively, in their hosts’ apoplast (Bayram and Braus, 2012; Müller et al., 2018). Beneficial microbes also lower the apoplastic pH to establish their interactions, as reported for *Trichoderma atroviride* (Pelagio-Flores et al., 2017), where fungal and plant AHAs participate in the acidification of the apoplast (López-Coria et al., 2016; Pelagio-Flores et al., 2017).

On the other hand, some microbes benefit from an alkalized apoplast, which they trigger during infection. This is the case of various fruit-infecting microbes like *Colletotrichum spp*. and *Alternaria alternata* (Prusky et al., 2001; Alkan et al., 2013). However, the reality is more complex as the same pathogen can induce either acidification or alkalization during the process of host infection, and apoplast acidification also impacts plant defense (Kesten et al., 2019; Westphal et al., 2019). Those fruit pathogens seem to secrete small pH modulators that increase or decrease the environmental pH depending on the availability of carbon, with acidification occurring under carbon excess (Bi et al., 2016). Similarly, *F. oxysporum* has recently been reported to induce an immediate acidification of the apoplast (Kesten et al., 2019), followed by alkalinization over time (Masachis et al., 2016; Kesten et al., 2019).

*Fusarium* spp. rely on an alkaline environment to infect the plants (Masachis et al., 2016; Wood et al., 2020), which they achieve by secreting small peptides that mimic plant rapid alkalinization factors (RALFs; Masachis et al., 2016). Plant RALFs have been shown to bind to the CW-bound leucine-rich repeat extensins (LRXs), potentially influencing the CW properties (Mecchia et al., 2017; Fabrice et al., 2018; Herger et al., 2019). A similar interaction can be envisioned for the pathogen RALF-likes which could alter the plant CW properties. The mechanism used by *F. oxysporum* to induce the initial apoplast acidification, how it modulates pH inside the root and how the molecular switch between acidification and alkalinization occurs are unknown. In addition, the dramatic and immediate apoplastic pH drop observed upon *F. oxysporum* contact impairs the cellulose synthesis machinery through the removal of CSCs from the plasma membrane and disassembly of the cortical microtubules, which influences the plant–fungal interaction (Figure 2D; Kesten et al., 2019; Menna et al., 2021). The changes in the apoplastic pH and their influence on the result of the plant–microbe interaction are time, organ, and even cell layer dependent. The complexity of apoplastic pH changes during plant–microbe interaction needs to be untangled with greater spatiotemporal resolution in different pathosystems. In addition, it will be essential to unravel the contribution of plant and pathogen proteins to these pH changes, at each stage of the interaction.

Apoplastic pH not only influences the development of disease, but it also has relevant effects on the plant CW, altering its physical and chemical properties. As mentioned above in “The plant CW”, the activity of many CW proteins is pH regulated, such as expansins, XTH/XETs, PMEs, and PMEIs (Figure 2C). Thus the drop in pH might enhance CW loosening by expansins, shift the balance between hydrolysis and cross-linking by transglycosylation or increase the processive generation of blocks of demethylated HG residues (Figure 2, A and B; McQueen-Mason et al., 1993; Hocq et al., 2021; Sampedro and Cosgrove, 2005). Also, the apoplastic pH influences the binding of cellulose and pectin (Figure 2C) nonenzymatically (Phyo et al., 2019). Thus, microbial modulation of apoplast pH might alter the activity of both plant and microbial CWDE and CW modifying proteins, and may modify polysaccharide interactions, which could enable a CW restructuring that favors colonization.

### CW ions: Ca^2+^ and B

The detection of CW modification results in changes in cell signaling, particularly involving apoplastic ions. Among them, Ca^2+^ ions are some of the first actors in PTI, working as a secondary messenger that regulates the coordination of plant defense or adaptation to the presence of the microbe (Ranty et al., 2016; Zipfel and Oldroyd, 2017). Ca^2+^ is transported from the apoplast to the cytosol through plasma membrane-localized cyclic nucleotide-gated calcium channels at the plasma membrane, opened upon phosphorylation by active PRRs (He et al., 2019; Tian et al., 2019). In addition, Ca^2+^ can enter the cytosol from organelles, especially the vacuole (Hilleary et al., 2020). The participation of other channels and pumps in this Ca^2+^-influx and their contribution to microbe-triggered plant responses remains to be fully investigated. Once Ca^2+^ is inside the cytosol, the plant-specific family of Ca^2+^-dependent protein kinases (CDPKs) function as relays to decode Ca^2+^ signals. CDPKs have different affinities for Ca^2+^ and thereby the downstream signaling distinguishes between various Ca^2+^-stimuli, as reviewed before (Boudsocq and Sheen, 2013; Yip Delormel and Boudsocq, 2019).

Apoplastic Ca^2+^ interacts with plant CW components containing charged uronic acids (GlcA or GalA); i.e. demethylated-HG and AGPs, shaping the structure and rigidity of the CW, as discussed in the “The plant CW” (Figure 1, C and D). This pH-sensitive pool of Ca^2+^ may be the origin of part of the Ca^2+^-influx from the apoplast and might have important amplificatory effects on microbe-induced Ca^2+^-signals (Figure 2E). Supporting this, the loss of AGP GlcA-residues impairs plant development and alters Ca^2+^-signaling in Arabidopsis in response to H_2_O_2_ treatment, but the effect of such mutants on Ca^2+^-signaling in the context of plant–microbe interactions has not been investigated (Lopez-Hernandez et al., 2020).

In addition to being a Ca^2+^ store, the Ca^2+^-binding by AGP might enable their pH-regulatable multimerization and/or cross-linking to other polysaccharides (Tryfona et al., 2012; Lamport and Várnai, 2013). If the Ca^2+^–AGP interaction contributes to mechanical stability of the plasma membrane and/or the CW, the pH changes may affect this property. The plasma membrane is an important mechanosensing part of the cell, containing haptosensitive channels, thus pH-induced Ca^2+^-release from CW polysaccharides may be amplified by the opening of these channels (Codjoe et al., 2022). Interestingly, classical AGPs (Schultz et al., 2002), tend to be upregulated in various pathosystems including bacterial and fungal hemibiotrophic vascular pathogens, such as *Fusarium* spp. and *Ralstonia* spp., and a fungal biotroph *Erisyphe necator* (Hu et al., 2008; Menna et al., 2021; Pimentel et al., 2021) but not in a bacterial biotrophic pathogen of brassicas, *Plasmodiophora brassicae* (Badstöber et al., 2020). It is unknown how much of the internalized Ca^2+^ is soluble or CW bound in the apoplast and if the release of CW-bound Ca^2+^ alone is sufficient to trigger the Ca^2+^-peak and downstream signaling.

Microbes attempt to minimize plant Ca^2+^-influxes to establish their desired interaction with the host (Figure 2E). The bacterial CW component xanthan, a negatively charged polymer, is an efficient Ca^2+^ chelator in vitro (Aslam et al., 2008) that can suppress Arabidopsis immunity (Yun et al., 2006; Aslam et al., 2008). Ca^2+^ and other divalent cations play a substantial role in the attachment of *Rhizobium leguminosarum* to root hairs. The acidic extracellular polymeric substances (EPSs) of these bacteria chelate the apoplastic Ca^2+^, allowing for the gelification of EPSs that act as “glue” that attaches the microbes to the plant surface (Morris et al., 1989; Williams et al., 2008). Although not fully understood, Ca^2+^ plays a role in the attachment of *Agrobacterium tumefaciens* to roots and infection thereof (Matthysse, 2014). In addition to Ca^2+^-binding by microbial polysaccharides, a Ca^2+^-binding protein, rhicadhesin, participates in nonspecific attachment of bacteria to roots (Smit et al., 1992; Denny, 1995). In summary, the dampening of Ca^2+^-signaling seems to serve an important function in suppressing plant PTI in both pathogenic and mutualistic interactions.

Together with Ca^2+^, boron also has an important structural function in plant CWs. In the form of borate esters, boron interconnects RG-II (Ishii et al., 1999; Höfte and Voxeur, 2017) and might be involved in linking glycans of AGPs and extensins with each other (Tan et al., 2018). In vitro, the stability of borate esters between AGPs/extensins is also regulated by pH. Boron deficiency leads to structural damage and limits plant growth by leading to a “swollen” CW (Wimmer and Eichert, 2013; Novaković et al., 2018). Recently, boron accumulation in the soil was shown to negatively impact microbial diversity, affecting bacteria more than fungi (Vera et al., 2021). Whether plant CW boron plays a direct role in plant–microbe interactions has not been studied so far, but the effects that boron insufficiency and overaccumulation have on the plant and the microbes in soil suggests that this molecule might influence the interactions.

## Plant CW reinforcement for defense

### CW polysaccharides

Modification and de novo synthesis of certain polysaccharides are part of the plant response to microbe colonization. Callose synthesis is especially important in the formation of papillae in response to biotrophic pathogens (Figure 2F) (Bellincampi et al., 2014; Wang et al., 2021b), while some fungi considered hemibiotrophs do not induce this plant defense, in some hosts (Carella et al., 2019; Menna et al., 2021a). The papillae contain additional components including cellulose, xylan, and antimicrobial peptides and metabolites (Wang et al., 2021a). It is not clear why callose might provide physical reinforcement to the CW, because in vitro mixtures suggest that callose reduces the stiffness of cellulose and increases ductility (Abou-Saleh et al., 2018). This callose-induced alteration in material behavior might be useful in preventing brittle breaks in the CW arising from the high pressure of appressoria. Due to the shape of the β-1,3 backbone of callose, it is unlikely to interact strongly with other CW components such as cellulose. It has also been suggested that callose fills pores in the CW, which might limit penetration of CWDEs or effectors into the CW (Eggert et al., 2014).

In addition, callose may have nonstructural roles. It could have a masking effect on the plant CW, especially against fungal pathogens. Fungal CWs contain β-1,3-glucans, and thus a high concentration of β-1,3-glucans from callose next to fungal hyphae may cause fungi to perceive the plant as “self” and downregulate virulence factor expression and/or secretion. The CW integrity and stress response component domain, reported to bind to polysaccharides including xylan and β-1,3-glucan, is found in fungal cell surface receptors that appear to be important for their development (Verna et al., 1997; Oide et al., 2019). In addition, the fungal CW integrity sensing pathway regulates the expression of genes, including secondary metabolite synthesis, required for fungal disease (Valiante, 2017). Thus, the direct sensing of β-1,3-glucans from callose may affect fungal behavior, though this speculative idea needs to be tested.

Together with the activation of the callose deposition machinery, several other plant polysaccharide synthesis gene families are transcriptionally upregulated in response to infections (Carella et al., 2019; Menna et al., 2021). Notably, in some cases, the CW components that are affected have not been confirmed yet. For example, a liverwort increased the expression of two enzymes probably involved in the synthesis of xyloglucan and xylan in response to an oomycete (Carella et al., 2019). Enzymes with no predicted function are also upregulated in some infected plants, particularly of the cellulose synthase-like (CSL) Family. Arabidopsis roots infected with *F. oxysporum* increase the expression of a *CSLE* gene family member (Menna et al., 2021). Related *CSLEs* are also upregulated in Brassicas with clubroot disease and powdery mildew-infected grapes (Badstöber et al., 2020; Pimentel et al., 2021), while sweet orange (*Citrus sinensis*) upregulated a *CSLB* and a *CSLE* in response to a bacterium–virus co-infection (Fu et al., 2017). The expression of another *CSL* gene, *AtCSLG2*, increases in Arabidopsis attacked by *R. solanacearum* (Hu et al., 2008). The products of these CSLE, CSLB, and CSLG family members are not known, but other related CSL families have the capacity to synthesize mixed linkage glucans with β-1,3- and β-1,4-glycosidic bonds and mannans (Little et al., 2018). Mixed linkage glucans have not been reported to exist in eudicots, but these CSL sub-families could synthesize specialized cellulose, mannans, callose, or a hitherto undiscovered polysaccharide, in response to pathogen infection. The overexpression of these enzymes, coupled with NMR, linkage and monosaccharide composition analysis will help us to discover their products and their role in plant defense.

Plant CW modifying proteins are also transcriptionally upregulated in response to pathogen infection, particularly CWDEs. For instance, in the interaction between *F. oxysporum* and Arabidopsis, the plant upregulates some pectate lyases, pectinases, methyl and acetyl esterases, and XTH/XETs (Menna et al., 2021). Cotton upregulates a pectinase during *V. dahliae* infection; a liverwort upregulates a predicted XTH/XET and an expansin when infected by an oomycete, and a plant XTH/XET is upregulated during *R. solanacearum* infection of Arabidopsis (Carella et al., 2019; Menna et al., 2021; Xiong et al., 2021). In principle, these endogenous enzymes have similar activities to the pathogen CWDEs, and thus can act as plant susceptibility factors, as reported for tomato (*Solanum lycopersicum*) polygalacturonases, expansins, and glucanases; an Arabidopsis expansin and an orange XTH (Flors et al., 2007; Cantu et al., 2009; Molina et al., 2021).

Plant CWDEs can also reinforce the host CW or boost the effectiveness of DAMPs, increasing the plant resistance. For example, the upregulation of an XTH in a resistant jute (*Corchorus trilocularis*) variety during infection with *Macrophomina phaseolina* may reinforce the CW by cross-linking (Sharmin et al., 2012). As pectin degradation relies on its degree of methylesterification, PME and PMEIs influence the generation of OGs. In addition, PMEs have been reported to demethylate OGs, enhancing their capacity to activate defense responses (Sharmin et al., 2012; Xue et al., 2021). At the same time, the plant can also synthesize proteinaceous inhibitors to reduce the activity of its own and the microbial CWDEs. Among them, PMEIs reduce the demethylation of pectins by PMEs, positively enhancing disease resistance (Lionetti et al., 2017; An et al., 2008; Liu et al., 2018). The contribution of plant CWDEs and their inhibitors to the plant response to microbes will need to be investigated further and more thoroughly linked to their specific biochemical activities, which will themselves likely be dynamic and regulated by pH and other apoplastic conditions (see the “Apoplastic pH” and “CW ions”).

### ROS and lignin

Another essential element of plant apoplastic defense against microbes is the production of ROS by the action of plasma membrane-localized NADPH-oxidases, named respiratory burst oxidase homologs (RBOHs), and CW peroxidases (Torres, 2010; Daudi et al., 2012; Survila et al., 2016). ROS include highly reactive molecules including superoxide (O_2_^−^), hydrogen peroxide (H_2_O_2_), hydroxyl radicals (OH^.^), and singlet oxygen (^1^O_2_) (Apel and Hirt, 2004; Sharma et al., 2012; Janků et al., 2019). ROS act as toxic molecules to fight invading microbes and as signaling molecules to activate plant defense (Castro et al., 2021). In addition, ROS participate in physically blocking the intruder path by CW strengthening via the induction of callose synthesis genes and spatially restricted lignin deposition (Wang et al., 2021c).

Lignin is the product of the oxidative polymerization of three main monolignols, synthesized in the cytosol through the general phenylpropanoid biosynthetic pathway and secreted to the apoplast. Once at the CW, those monolignols are activated by the oxidation systems peroxidase/H_2_O_2_ and/or laccase/O_2_ to be assembled into the final lignin polymers (Tobimatsu and Schuetz, 2019; Mnich et al., 2020). Lignification is a common response of plants to microbe infection (Figure 2G), starting with the triggering of the phenylpropanoid synthesis pathway (Ishihara et al., 2012; Galindo-González and Deyholos, 2016; Novo et al., 2017; Ferreira et al., 2017; Sabella et al., 2018). The monolignol polymerization can occur in highly restricted areas around pathogen cells, as shown in the Arabidopsis–*Pseudomonas syringae* pv *tomato* interaction, where localized lignification surrounding the bacteria reduced their movement (Lee et al., 2019). Consistently, more virulent bacteria strains possess effectors to suppress a strong ROS burst and lignification (Lee et al., 2019).

The highly contained formation of lignin seems to be a consequence of the precise localization and action of specific proteins. This event is well studied during the formation of the Casparian strip, where CASP proteins form complexes with peroxidases and RBOHs in small domains of the plasma membrane (Barbosa et al., 2019). The co-localization of peroxidases, and the RBOHs that produce their peroxide fuel, allows the formation of the thin strip of lignin (Barbosa et al., 2019; Rojas-Murcia et al., 2020). Pathogen-induced lignification may be analogous to Casparian strip formation. Indeed, the deposition of lignin around pathogenic bacteria in Arabidopsis depends on two CASP-like (CASPL) proteins that localize in the plant plasma membrane surrounding the newly formed lignin (Lee et al., 2019). Binding to CASPLs is not the only mechanism for the specific localization of peroxidases and laccases within the CW (Hoffmann et al., 2020). Some peroxidases have been shown to bind to demethylated HG, and this can restrict them to specific regions of the CW (Figure 2G; Francoz et al., 2019; Shah et al., 2004). A similar mechanism can be envisioned during microbe-induced HG-demethylation, leading to the localization of certain peroxidases required for lignin formation to isolate the microbe and/or to protect the pectin regions that are more vulnerable to degradation by the intruder. This and other hypotheses remain to be tested, like the capacity of peroxidases to bind to other polysaccharide epitopes, or the possibility of laccases to have a similar polysaccharide-binding capacity.

Peroxidases also contribute to plant defense by cross-linking structural proteins in the CW, such as extensins and AGPs (Almagro et al., 2009; Mishler-Elmore et al., 2021), whose role in plant–microbe interactions was recently reviewed (Castilleux et al., 2018, 2021; Mishler-Elmore et al., 2021). In addition, other peroxidases synthesize ROS from the RBOH-derived H_2_O_2_ (Almagro et al., 2009; Tobimatsu and Schuetz, 2019). Thus, regulating peroxidase activity may be a mechanism for pathogens to reduce CW reinforcement and other plant defenses. This can be achieved by controlling apoplastic pH, as pH influences the activity of peroxidases and laccasses (Pedreño et al., 1989; Nunes and Kunamneni, 2018). Moreover, the influence of pH on RBOHs, which provide the required ROS to peroxidases and laccases, is predicted but not fully understood. In addition, the influence of high proton concentration on the activity of ROS molecules remains to be shown.

Peroxidase activity can be further controlled by calcium ions; as it has been proposed that a drop in apoplastic Ca^2+^ may act as an off-switch for peroxidase activity (Plieth, 2012). Thus, when Ca^2+^ is rapidly internalized by the plant cells upon microbe attack, peroxidase activity could be limited (Hemetsberger et al., 2012). Some laccases include predicted calmodulin domains (Zhang et al., 2019), indicating that they may also be regulated by Ca^2+^, though the exact role of these domains remains unknown. Lignin deposition and other ROS-dependent plant immune responses can also be minimized by the microbe through secretion of proteins that interfere with the host ROS-generating system, like the effector PEP1 of *Ustilago maydis* (Hemetsberger et al., 2012; Navarrete et al., 2021). Other microbes have evolved mechanisms to sequester ROS, such as the EPS of the bacteria *Azorhizobium caulinodans*, that sequesters H_2_O_2_ allowing the formation of root nodules in *Sesbiana rostrata* (Figure 2G; D’Haeze et al., 2004). The opportunistic fungal pathogen *Alternaria tenuissima* produces an EPS with a high potential for scavenging hydroxyl radicals and superoxide anions in vitro (Wang et al., 2019), though the in planta function remains to be tested. Recently, fungal endophytes and pathogens were shown to exploit a plant GH to release a conserved oligosaccharide from their EPS, that acts as a ROS scavenger and subvert plant immune responses (Chandrasekar et al., 2021). In addition, acidification of the apoplast or the rhizosphere by microbes decreases the ROS generation potential of the plant (Yu et al., 2019).

### Suberin

Together with lignin, suberin forms another layer of nonpolysaccharide protection against pathogen entrance into individual plant cells. It constitutes a hydrophobic barrier that naturally occurs in specialized tissues, such as seed coats, leaf epidermal cells, and periderm, or is specifically formed after wounding and microbe attack (Ranathunge et al., 2008; Philippe et al., 2020; Singh et al., 2021). Suberin monomers seem to be secreted through vesicular-tubular membrane structures that fuse to the plasma membrane and release their cargo (De Bellis et al., 2021). Once outside the cell, suberin becomes polymerized and forms a lamellar sheet between the plasma membrane and the CW (Philippe et al., 2020).

Suberization seems to be induced mainly by vascular pathogens to effectively limit their spread between vascular bundles and keep them contained in the already infected tissue (Robb et al., 1991; Kashyap et al., 2021b). In the interaction between *Vitis vinifera* and the vascular wilt pathogen *Phaeomoniella chlamydospora*, suberin deposition was shown to be active as a limiting factor for pathogen spread between xylem vessels (Pouzoulet et al., 2017). Similarly, the deposition of a ligno-suberin coating and tyramine-derived hydroxycinnamic acid amides have recently been reported to be required for tomato resistance to *R. solanacearum* (Kashyap et al., 2021a). Suberin deposition upon vascular pathogen infection is positively regulated by the transcription factor MYB41, whose promoter is specifically induced during stress conditions and its overexpression leads to ectopic suberization of various plant species and tissue types (Kosma et al., 2014; Vishwanath et al., 2015).

The regulation of suberin deposition in the context of plant–microbe interactions probably requires additional transcription regulators. The recently described MYBs that, together with MYB41, are sufficient to promote endodermal suberin deposition are interesting candidates to induce the same process upon biotic stress, but this role has to be confirmed (Shukla et al., 2021). Also, it will be important to understand suberin assembly in the wall, which is likely to occur through transesterification by enzymes similar to cutin synthases (Philippe et al., 2020). Moreover, the degradation of suberin by microbes is not well understood during plant colonization, and will also need to be investigated further, though various suberinases have been identified (Fernando et al., 1984).

## Summary and perspectives

The plant CW is a dynamic, interactive part of the plant cell, whose properties change in response to the action of CW modifying enzymes, alterations in ionic composition, and the synthesis of new material. These changes are particularly active in the context of plant–microbe interactions, where both organisms modify the plant CW and its environment and, at different time points, the same organism may reverse earlier changes.

Thus far, research in the field of plant CW role in host–microbe interaction has largely focused on the functional importance of individual CWDEs in causing disease, the role of CWD products as DAMPs, and the synthesis of new CW material as a defense response. These remain key areas of research; for instance, new CW-derived DAMPs have been identified whose receptor is unknown, many plasma membrane receptors have been reported to affect disease resistance but their ligands and/or exact role in plant defense have not been characterized. Proteomic technologies may be useful in this area, such as spatial proteomics and phosphoproteomics, to identify receptors and signaling components which respond to disease or to specific signals. Also, the coordination of plant response to its own CW-derived DAMPs and microbial-generated ones is an essential topic to be further explored. The dynamic and subcellular nature of plant CW-related changes, polysaccharide and ionic, mean that it is necessary to monitor interactions with higher temporal and spatial resolution, such as in different cell layers and cell areas. This should be possible by adapting to various pathosystems, the high-resolution microscopy, and new probes currently available or under-development in the plant biology field. We will also need to investigate the structural and mechanical changes in the plant CW during plant growth and in response to microbe colonization using cryo-electron microscopy, solid-state NMR and atomic force microscopy, among other techniques. Integrating such knowledge with computational simulations will enable us to analyze how plant CW structure and composition relates to mechanical properties. At the same time, it will be important to biochemically characterize individual members of large gene families (e.g. expansins, XTHs, and CSLs) to understand their functions in specific plant–microbe interactions. As an essential component of the plant cell with vital roles in plant–microbe interactions, it is extremely important to continue investigating how CW structure and composition relates to its properties, and how these are modified in response to environmental stress and coordinated with plant development.

## Notes

The author responsible for distribution of materials integral to the findings presented in this article in accordance with the policy described in the Instructions for Authors (https://academic.oup.com/plcell) is: Clara Sánchez-Rodríguez (clara_sanchez@ethz.ch)

## References

[koac040-B1] Abou-Saleh RH , Hernandez-Gomez MC, Amsbury S, Paniagua C, Bourdon M, Miyashima S, Helariutta Y, Fuller M, Budtova T, Connell SD, et al (2018) Interactions between callose and cellulose revealed through the analysis of biopolymer mixtures. Nat Commun 9: 4538 30382102 10.1038/s41467-018-06820-y PMC6208431

[koac040-B2] Alkan N , Espeso EA, Prusky D (2013) Virulence regulation of phytopathogenic fungi by pH. Antioxid. Redox Signal 19: 1012–1025 23249178 10.1089/ars.2012.5062

[koac040-B3] Almagro L , Gómez Ros LV, Belchi-Navarro S, Bru R, Ros Barceló A, Pedreño MA (2009) Class III peroxidases in plant defence reactions. J Exp Bot 60: 377–390 19073963 10.1093/jxb/ern277

[koac040-B4] Altartouri B , Bidhendi AJ, Tani T, Suzuki J, Conrad C, Chebli Y, Liu N, Karunakaran C, Scarcelli G, Geitmann A (2019) Pectin chemistry and cellulose crystallinity govern pavement cell morphogenesis in a multi-step mechanism. Plant Physiol 181: 127–141 31363005 10.1104/pp.19.00303 PMC6716242

[koac040-B5] An SH , Sohn KH, Choi HW, Hwang IS, Lee SC, Hwang BK (2008). Pepper pectin methylesterase inhibitor protein CaPMEI1 is required for antifungal activity, basal disease resistance and abiotic stress tolerance. Planta 228: 61–78 18327607 10.1007/s00425-008-0719-z PMC2413075

[koac040-B6] Apel K , Hirt H (2004) Reactive oxygen species: metabolism, oxidative stress, and signal transduction. Annu Rev Plant Biol 55: 373–399 15377225 10.1146/annurev.arplant.55.031903.141701

[koac040-B7] Aslam SN , Newman MA, Erbs G, Morrissey KL, Chinchilla D, Boller T, Jensen TT, De Castro C, Ierano T, Molinaro A, et al (2008). Bacterial polysaccharides suppress induced innate immunity by calcium chelation. Curr Biol 18: 1078–1083 18639458 10.1016/j.cub.2008.06.061

[koac040-B8] Bacete L , Hamann T (2020) The role of mechanoperception in plant cell wall integrity maintenance. Plants 9: 574 10.3390/plants9050574 PMC7285163 32369932

[koac040-B9] Bacete L , Mélida H, Miedes E, Molina A (2018) Plant cell wall-mediated immunity: cell wall changes trigger disease resistance responses. Plant J 93: 614–636 29266460 10.1111/tpj.13807

[koac040-B10] Badstöber J , Ciaghi S, Neuhauser S (2020) Dynamic cell wall modifications in brassicas during clubroot disease. bioRxiv: 2020.03.02.972901

[koac040-B11] Balestrini R , Bonfante P (2014) Cell wall remodeling in mycorrhizal symbiosis: a way towards biotrophism. Front Plant Sci 5: 237 24926297 10.3389/fpls.2014.00237 PMC4044974

[koac040-B12] Barbosa ICR , Rojas-Murcia N, Geldner N (2019) The Casparian strip-one ring to bring cell biology to lignification? Curr Opin Biotechnol 56: 121–129 30502636 10.1016/j.copbio.2018.10.004

[koac040-B13] Barghahn S , Arnal G, Jain N, Petutschnig E, Brumer H, Lipka V (2021) Mixed linkage β-1,3/1,4-glucan oligosaccharides induce defense responses in *Hordeum vulgare* and *Arabidopsis thaliana*. Front Plant Sci 12, https://doi.org/10.3389/fpls.2021.682439 10.3389/fpls.2021.682439 PMC8247929 34220903

[koac040-B14] Basaran P , Ozcan M, Denisov Y, Freeman S (2007) Elucidation of pectinolytic enzyme activities of a non-pathogenic watermelon pathogen mutant, Fusarium oxysporum f.sp. niveum M87. Australas Plant Pathol 36: 135

[koac040-B15] Bayram O , Braus GH (2012) Coordination of secondary metabolism and development in fungi: the velvet family of regulatory proteins. FEMS Microbiol Rev 36: 1–24 21658084 10.1111/j.1574-6976.2011.00285.x

[koac040-B16] Bellincampi D , Cervone F, Lionetti V (2014) Plant cell wall dynamics and wall-related susceptibility in plant-pathogen interactions. Front Plant Sci 5: 228 24904623 10.3389/fpls.2014.00228 PMC4036129

[koac040-B17] Benedetti M , Verrascina I, Pontiggia D, Locci F, Mattei B, De Lorenzo G, Cervone F (2018) Four Arabidopsis berberine bridge enzyme-like proteins are specific oxidases that inactivate the elicitor-active oligogalacturonides. Plant J 94: 260–273 29396998 10.1111/tpj.13852

[koac040-B18] Benschop JJ , Mohammed S, O’Flaherty M, Heck AJR, Slijper M, Menke FLH (2007) Quantitative phosphoproteomics of early elicitor signaling in Arabidopsis. Mol Cell Proteomics 6: 1198–1214 17317660 10.1074/mcp.M600429-MCP200

[koac040-B19] Bi F , Barad S, Ment D, Luria N, Dubey A, Casado V, Glam N, Mínguez JD, Espeso EA, Fluhr R, et al (2016) Carbon regulation of environmental pH by secreted small molecules that modulate pathogenicity in phytopathogenic fungi. Mol Plant Pathol 17: 1178–1195 26666972 10.1111/mpp.12355 PMC6638356

[koac040-B20] Bizuneh GK (2021) The chemical diversity and biological activities of phytoalexins. Adv Tradit Med 21: 31–43

[koac040-B21] Bosch M , Cheung AY, Hepler PK (2005) Pectin methylesterase, a regulator of pollen tube growth. Plant Physiol 138: 1334–1346 15951488 10.1104/pp.105.059865 PMC1176407

[koac040-B22] Bou Daher F , Chen Y, Bozorg B, Clough J, Jönsson H, Braybrook SA (2018) Anisotropic growth is achieved through the additive mechanical effect of material anisotropy and elastic asymmetry. eLife 7: e38161 10.7554/eLife.38161 PMC6143341 30226465

[koac040-B23] Boudsocq M , Sheen J (2013) CDPKs in immune and stress signaling. Trends Plant Sci 18: 30–40 22974587 10.1016/j.tplants.2012.08.008 PMC3534830

[koac040-B24] Burton RA , Gidley MJ, Fincher GB (2010) Heterogeneity in the chemistry, structure and function of plant cell walls. Nat Chem Biol 6: 724–732 20852610 10.1038/nchembio.439

[koac040-B25] Cantu D , Blanco-Ulate B, Yang L, Labavitch JM, Bennett ABP, Ann L (2009) Ripening regulated susceptibility of tomato fruit to Botrytis cinerea requires NOR but not RIN or ethylene. Plant Physiol 150: 1434–1449 19465579 10.1104/pp.109.138701 PMC2705034

[koac040-B26] Carella P , Gogleva A, Hoey DJ, Bridgen AJ, Stolze SC, Nakagami H, Schornack S (2019) Conserved biochemical defenses underpin host responses to oomycete infection in an early-divergent land plant lineage. Curr Biol 29: 2282–2294.e5 31303485 10.1016/j.cub.2019.05.078

[koac040-B27] Castilleux R , Plancot B, Ropitaux M, Carreras A, Leprince J, Boulogne I, Follet-Gueye M-L, Popper ZA, Driouich A, Vicré M (2018) Cell wall extensins in root-microbe interactions and root secretions. J Exp Bot 69: 4235–4247 29945246 10.1093/jxb/ery238

[koac040-B28] Castilleux R , Plancot B, Vicré M, Nguema-Ona E, Driouich A (2021) Extensin, an underestimated key component of cell wall defence? Ann Bot 127: 709–713 33723574 10.1093/aob/mcab001 PMC8103801

[koac040-B29] Castro B , Citterico M, Kimura S, Stevens DM, Wrzaczek M, Coaker G (2021) Stress-induced reactive oxygen species compartmentalization, perception and signalling. Nat Plants 7: 403–412 33846592 10.1038/s41477-021-00887-0 PMC8751180

[koac040-B30] Chandrasekar B , Wanke A, Wawra S, Saake P, Mahdi L, Charura N, Neidert M, Malisic M, Thiele M, Dama M, et al (2021) Fungi hijack a plant apoplastic endoglucanase to release a ROS scavenging β-glucan decasaccharide to subvert immune responses. bioRxiv: 2021.05.10.443455 10.1093/plcell/koac114 PMC9252488 35441693

[koac040-B31] Claverie J , Balacey S, Lemaître-Guillier C, Brulé D, Chiltz A, Granet L, Noirot E, Daire X, Darblade B, Héloir M-C, et al (2018) The cell wall-derived xyloglucan is a new DAMP triggering plant immunity in *Vitis vinifera* and *Arabidopsis thaliana*. Front Plant Sci 9: 1725 30546374 10.3389/fpls.2018.01725 PMC6280107

[koac040-B32] Coaker SLDS (2020) Plant NLR-triggered immunity: from receptor activation to downstream signaling. Curr Opin Immunol 62: 99–105 31958770 10.1016/j.coi.2019.12.007 PMC7190197

[koac040-B33] Codjoe JM , Miller K, Haswell ES (2022) Plant cell mechanobiology: greater than the sum of its parts. Plant Cell 34: 129–145 34524447 10.1093/plcell/koab230 PMC8773992

[koac040-B34] Cosgrove DJ (2014) Re-constructing our models of cellulose and primary cell wall assembly. Curr Opin Plant Biol 22: 122–131 25460077 10.1016/j.pbi.2014.11.001 PMC4293254

[koac040-B35] Cosgrove DJ , Anderson CT (2020) Plant cell growth: do pectins drive lobe formation in Arabidopsis pavement cells? Curr Biol 30: R660–R662 32516619 10.1016/j.cub.2020.04.007

[koac040-B36] Daudi A , Cheng Z, O’Brien JA, Mammarella N, Khan S, Ausubel FM, Bolwell GP (2012) The apoplastic oxidative burst peroxidase in Arabidopsis is a major component of pattern-triggered immunity. Plant Cell 24: 275–287 22247251 10.1105/tpc.111.093039 PMC3289579

[koac040-B37] De Bellis D , Kalmbach L, Marhavy P, Daraspe J, Geldner N, Barberon M (2021) Extracellular membrane tubules involved in suberin deposition in plant cell walls. bioRxiv: 2021.02.02.429332 10.1038/s41467-022-29110-0 PMC8933581 35304458

[koac040-B38] DeFalco TA , Zipfel C (2021) Molecular mechanisms of early plant pattern-triggered immune signaling. Mol Cell 81: 3449–3467 34403694 10.1016/j.molcel.2021.07.029

[koac040-B39] Denny TP (1995) Involvement of bacterial polysaccharides in plant pathogenesis. Annu Rev Phytopathol 33: 173–197 18999958 10.1146/annurev.py.33.090195.001133

[koac040-B40] Desaki Y , Kouzai Y, Ninomiya Y, Iwase R, Shimizu Y, Seko K, Molinaro A, Minami E, Shibuya N, Kaku H, et al (2018) OsCERK1 plays a crucial role in the lipopolysaccharide-induced immune response of rice. New Phytol 217: 1042–1049 29194635 10.1111/nph.14941

[koac040-B41] D’Haeze W , Glushka J, De Rycke R, Holsters M, Carlson RW (2004) Structural characterization of extracellular polysaccharides of Azorhizobium caulinodans and importance for nodule initiation on Sesbania rostrata. Mol Microbiol 52: 485–500 15066035 10.1111/j.1365-2958.2004.03989.x

[koac040-B42] de Azevedo Souza C , Li S, Lin AZ, Boutrot F, Grossmann G, Zipfel C, Somerville SC (2017) Cellulose-derived oligomers act as damage-associated molecular patterns and trigger defense-like responses. Plant Physiol 173: 2383–2398 28242654 10.1104/pp.16.01680 PMC5373054

[koac040-B43] Dick-Pérez M , Zhang Y, Hayes J, Salazar A, Zabotina OA, Hong M (2011) Structure and interactions of plant cell-wall polysaccharides by two- and three-dimensional magic-angle-spinning solid-state NMR. Biochemistry 50: 989–1000 21204530 10.1021/bi101795q

[koac040-B44] Diehl BG , Brown NR (2014) Lignin cross-links with cysteine- and tyrosine-containing peptides under biomimetic conditions. J Agric Food Chem 62: 10312–10319 25275918 10.1021/jf503897n

[koac040-B45] Eggert D , Naumann M, Reimer R, Voigt CA (2014) Nanoscale glucan polymer network causes pathogen resistance. Sci Rep 4: 4159 24561766 10.1038/srep04159 PMC3932449

[koac040-B46] Engelsdorf T , Gigli-Bisceglia N, Veerabagu M, McKenna JF, Vaahtera L, Augstein F, Van der Does D, Zipfel C, Hamann T (2018) The plant cell wall integrity maintenance and immune signaling systems cooperate to control stress responses in *Arabidopsis thaliana*. Sci Signal 11: eaao3070 10.1126/scisignal.aao3070 29945884

[koac040-B47] Escudero V , Jordá L, Sopeña-Torres S, Mélida H, Miedes E, Muñoz-Barrios A, Swami S, Alexander D, McKee LS, Sánchez-Vallet A, et al (2017) Alteration of cell wall xylan acetylation triggers defense responses that counterbalance the immune deficiencies of plants impaired in the β-subunit of the heterotrimeric G-protein. Plant J 92: 386–399 28792629 10.1111/tpj.13660 PMC5641240

[koac040-B48] Fabrice TN , Vogler H, Draeger C, Munglani G, Gupta S, Herger AG, Knox P, Grossniklaus U, Ringli C (2018) LRX proteins play a crucial role in pollen grain and pollen tube cell wall development. Plant Physiol 176: 1981–1992 29247121 10.1104/pp.17.01374 PMC5841697

[koac040-B49] Fernando G , Zimmermann W, Kolattukudy PE (1984) Suberin-grown Fusarium solani f. sp pisi generates a cutinase-like esterase which depolymerizes the aliphatic components of suberin. Physiol Plant Pathol 24: 143–155

[koac040-B50] Ferreira V , Pianzzola MJ, Vilaró FL, Galván GA, Tondo ML, Rodriguez MV, Orellano EG, Valls M, Siri MI (2017) Interspecific potato breeding lines display differential colonization patterns and induced defense responses after infection. Front Plant Sci 8: 1424 28894453 10.3389/fpls.2017.01424 PMC5581342

[koac040-B51] Flors V , Leyva Mde L, Vicedo B, Finiti I, Real MD, García-Agustín P, Bennett AB, González-Bosch C (2007) Absence of the endo-beta-1,4-glucanases Cel1 and Cel2 reduces susceptibility to Botrytis cinerea in tomato. Plant J 52: 1027–1040 17916112 10.1111/j.1365-313X.2007.03299.x

[koac040-B52] Francoz E , Ranocha P, Le Ru A, Martinez Y, Fourquaux I, Jauneau A, Dunand C, Burlat V (2019) Pectin demethylesterification generates platforms that anchor peroxidases to remodel plant cell wall domains. Dev Cell 48: 261–276.e8 30555001 10.1016/j.devcel.2018.11.016

[koac040-B53] Franková L , Fry SC (2021) Hemicellulose-remodelling transglycanase activities from charophytes: towards the evolution of the land-plant cell wall. Plant J 108: 7–28 34547150 10.1111/tpj.15500

[koac040-B54] Fu S , Shao J, Paul C, Zhou C, Hartung JS (2017) Transcriptional analysis of sweet orange trees co-infected with “Candidatus Liberibacter asiaticus” and mild or severe strains of Citrus tristeza virus. BMC Genomics 18: 1–17 29089035 10.1186/s12864-017-4174-8 PMC5664567

[koac040-B55] Galindo-González L , Deyholos MK (2016) RNA-seq transcriptome response of flax (L.) to the pathogenic fungus f. sp. Front Plant Sci 7: 1766 27933082 10.3389/fpls.2016.01766 PMC5121121

[koac040-B56] Gámez-Arjona FM , Vitale S, Voxeur A, Dora S, Müller S, Sancho-Andrés G, Montesinos JC, Di Pietro A, Sánchez-Rodríguez C (2021) Impairment of the cellulose degradation machinery enhances fungal virulence but limits reproductive fitness. bioRxiv: 2021.10.08.463612 10.1126/sciadv.abl9734 PMC9020665 35442735

[koac040-B57] Georgelis N , Nikolaidis N, Cosgrove DJ (2015) Bacterial expansins and related proteins from the world of microbes. Appl Microbiol Biotechnol 99: 3807–3823 25833181 10.1007/s00253-015-6534-0 PMC4427351

[koac040-B58] Gorshkova T , Mokshina N, Chernova T, Ibragimova N, Salnikov V, Mikshina P, Tryfona T, Banasiak A, Immerzeel P, Dupree P, et al (2015). Aspen tension wood fibers contain β-(1–-> 4)-galactans and acidic arabinogalactans retained by cellulose microfibrils in gelatinous walls. Plant Physiol 169: 2048–2063 26378099 10.1104/pp.15.00690 PMC4634055

[koac040-B59] Grantham NJ , Wurman-Rodrich J, Terrett OM, Lyczakowski JJ, Stott K, Iuga D, Simmons TJ, Durand-Tardif M, Brown SP, Dupree R, et al (2017). An even pattern of xylan substitution is critical for interaction with cellulose in plant cell walls. Nat Plants 3: 859–865 28993612 10.1038/s41477-017-0030-8

[koac040-B60] Gui Y-J , Chen J-Y, Zhang D-D, Li N-Y, Li T-G, Zhang W-Q, Wang X-Y, Short DPG, Li L, Guo W, et al (2017) *Verticillium dahliae* manipulates plant immunity by glycoside hydrolase 12 proteins in conjunction with carbohydrate-binding module 1. Environ Microbiol 19: 1914–1932 28205292 10.1111/1462-2920.13695

[koac040-B61] Ha CM , Rao X, Saxena G, Dixon RA (2021) Growth–defense trade-offs and yield loss in plants with engineered cell walls. New Phytol 231: 60–74 33811329 10.1111/nph.17383

[koac040-B62] Haas KT , Wightman R, Meyerowitz EM, Peaucelle A (2020) Pectin homogalacturonan nanofilament expansion drives morphogenesis in plant epidermal cells. Science 367: 1003–1007 32108107 10.1126/science.aaz5103 PMC7932746

[koac040-B63] Haddad Momeni M , Fredslund F, Bissaro B, Raji O, Vuong TV, Meier S, Nielsen TS, Lombard V, Guigliarelli B, Biaso F, et al (2021). Discovery of fungal oligosaccharide-oxidising flavo-enzymes with previously unknown substrates, redox-activity profiles and interplay with LPMOs. Nat Commun 12: 2132 33837197 10.1038/s41467-021-22372-0 PMC8035211

[koac040-B64] Hane JK , Paxman J, Jones DAB, Oliver RP, de Wit P (2020) “CATAStrophy,” a genome-informed trophic classification of filamentous plant pathogens – how many different types of filamentous plant pathogens are there? Front Microbiol 10: 3088 32038539 10.3389/fmicb.2019.03088 PMC6986263

[koac040-B65] Hemetsberger C , Herrberger C, Zechmann B, Hillmer M, Doehlemann G (2012) The Ustilago maydis effector Pep1 suppresses plant immunity by inhibition of host peroxidase activity. PLoS Pathog 8: e1002684 22589719 10.1371/journal.ppat.1002684 PMC3349748

[koac040-B66] Herger A , Dünser K, Kleine-Vehn J, Ringli C (2019) Leucine-rich repeat extensin proteins and their role in cell wall sensing. Curr Biol 29: R851–R858 31505187 10.1016/j.cub.2019.07.039

[koac040-B67] Hernández-Blanco C , Feng DX, Hu J, Sánchez-Vallet A, Deslandes L, Llorente F, Berrocal-Lobo M, Keller H, Barlet X, Sánchez-Rodríguez C, et al (2007). Impairment of cellulose synthases required for Arabidopsis secondary cell wall formation enhances disease resistance. Plant Cell 19: 890–903 17351116 10.1105/tpc.106.048058 PMC1867366

[koac040-B68] He Y , Zhou J, Meng X (2019) Phosphoregulation of Ca2+ Influx in Plant Immunity. Trends Plant Sci 24: 1067–1069 31668684 10.1016/j.tplants.2019.10.004

[koac040-B69] Hilleary R , Paez-Valencia J, Vens C, Toyota M, Palmgren M, Gilroy S (2020) Tonoplast-localized Ca2+ pumps regulate Ca2+ signals during pattern-triggered immunity in *Arabidopsis thaliana*. Proc Natl Acad Sci USA 117: 18849–18857 32690691 10.1073/pnas.2004183117 PMC7414185

[koac040-B70] Hocq L , Harbrylo O, Voxeur A, Roblot CP, Šafran J, Sénéchal F, Fournet F, Bassard S, Battu V, Demailly H, et al (2021). Arabidopsis AtPME2 has a pH-dependent processivity and control cell wall mechanical properties. bioRxiv: 2021.03.03.433777

[koac040-B71] Hoffmann N , Benske A, Betz H, Schuetz M, Samuels AL (2020) Laccases and peroxidases co-localize in lignified secondary cell walls throughout stem development. Plant Physiol 184: 806–822 32699027 10.1104/pp.20.00473 PMC7536695

[koac040-B72] Höfte H , Voxeur A (2017) Plant cell walls. Curr Biol 27: R865–R870 28898654 10.1016/j.cub.2017.05.025

[koac040-B73] Hou S , Liu Z, Shen H, Wu D (2019) Damage-associated molecular pattern-triggered immunity in plants. Front Plant Sci 10: 646 31191574 10.3389/fpls.2019.00646 PMC6547358

[koac040-B74] Huang JH , Kortstee A, Dees DC, Trindade LM, Visser RG, Gruppen H, Schols HA (2017) Evaluation of both targeted and non-targeted cell wall polysaccharides in transgenic potatoes. Carbohydr Polym 156: 312–321 27842828 10.1016/j.carbpol.2016.09.043

[koac040-B75] Huang Q , Allen C (1997) An exo-poly-alpha-D-galacturonosidase, PehB, is required for wild-type virulence of Ralstonia solanacearum. J Bacteriol 179: 7369–7378 9393701 10.1128/jb.179.23.7369-7378.1997 PMC179687

[koac040-B76] Huang Q , Allen C (2000) Polygalacturonases are required for rapid colonization and full virulence of Ralstonia solanacearum on tomato plants. Physiol Mol Plant Pathol 57: 77–83

[koac040-B77] Hu J , Barlet X, Deslandes L, Hirsch J, Feng DX, Somssich I, Marco Y (2008) Transcriptional responses of Arabidopsis thaliana during wilt disease caused by the soil-borne phytopathogenic bacterium, Ralstonia solanacearum. PLoS One 3: e2589 18596930 10.1371/journal.pone.0002589 PMC2435627

[koac040-B78] Hwang IS , Oh E-J, Lee HB, Oh C-S (2019) Functional characterization of two cellulase genes in the gram-positive pathogenic bacterium Clavibacter michiganensis for wilting in tomato. Mol Plant Microbe Interact 32: 491–501 30345870 10.1094/MPMI-08-18-0227-R

[koac040-B79] Ishihara T , Mitsuhara I, Takahashi H, Nakaho K (2012) Transcriptome analysis of quantitative resistance-specific response upon Ralstonia solanacearum infection in tomato. PLoS One 7: e46763 23071630 10.1371/journal.pone.0046763 PMC3465262

[koac040-B80] Ishii T , Matsunaga T, Pellerin P, O’Neill MA, Darvill A, Albersheim P (1999) The plant cell wall polysaccharide rhamnogalacturonan II self-assembles into a covalently cross-linked dimer. J Biol Chem 274: 13098–13104 10224062 10.1074/jbc.274.19.13098

[koac040-B81] Janků M , Luhová L, Petřivalský M (2019) On the origin and fate of reactive oxygen species in plant cell compartments. Antioxidants (Basel) 8: 105 10.3390/antiox8040105 PMC6523537 30999668

[koac040-B82] Jarvis MC , Apperley DC (1995) Chain conformation in concentrated pectic gels: evidence from ^13^C NMR. Carbohydr Res 275: 131–145

[koac040-B83] Jeworutzki E , Roelfsema MRG, Anschütz U, Krol E, Elzenga JTM, Felix G, Boller T, Hedrich R, Becker D (2010) Early signaling through the Arabidopsis pattern recognition receptors FLS2 and EFR involves Ca-associated opening of plasma membrane anion channels. Plant J 62: 367–378 20113440 10.1111/j.1365-313X.2010.04155.x

[koac040-B84] Kang X , Kirui A, Dickwella Widanage MC, Mentink-Vigier F, Cosgrove DJ, Wang T (2019) Lignin-polysaccharide interactions in plant secondary cell walls revealed by solid-state NMR. Nat Commun 10: 347 30664653 10.1038/s41467-018-08252-0 PMC6341099

[koac040-B85] Kashyap A , Capellades M, Zhang W, Srinivasan S, Laromaine A, Serra O, Figueras M, Rencoret J, Gutiérrez A, Valls M, et al (2021a). Induced ligno-suberin vascular coating and tyramine-derived hydroxycinnamic acid amides restrict Ralstonia solanacearum colonization in resistant tomato roots. bioRxiv: 2021.06.15.448549 10.1111/nph.17982 35152435

[koac040-B86] Kashyap A , Planas-Marquès M, Capellades M, Valls M, Coll NS (2021b) Blocking intruders: inducible physico-chemical barriers against plant vascular wilt pathogens. J Exp Bot 72: 184–198 32976552 10.1093/jxb/eraa444 PMC7853604

[koac040-B87] Kesten C , Gámez-Arjona FM, Menna A, Scholl S, Dora S, Huerta AI, Huang HY, Tintor N, Kinoshita T, Rep M, et al (2019) Pathogen-induced pH changes regulate the growth-defense balance in plants. EMBO J 38: e101822 31736111 10.15252/embj.2019101822 PMC6912046

[koac040-B88] King BC , Waxman KD, Nenni NV, Walker LP, Bergstrom GC, Gibson DM (2011) Arsenal of plant cell wall degrading enzymes reflects host preference among plant pathogenic fungi. Biotechnol Biofuels 4: 4 21324176 10.1186/1754-6834-4-4 PMC3051899

[koac040-B89] Kosma DK , Murmu J, Razeq FM, Santos P, Bourgault R, Molina I, Rowland O (2014) AtMYB41 activates ectopic suberin synthesis and assembly in multiple plant species and cell types. Plant J 80: 216–229 25060192 10.1111/tpj.12624 PMC4321041

[koac040-B90] Kostlánová N , Mitchell EP, Lortat-Jacob H, Oscarson S, Lahmann M, Gilboa-Garber N, Chambat G, Wimmerová M, Imberty A (2005) The fucose-binding lectin from Ralstonia solanacearum. A new type of beta-propeller architecture formed by oligomerization and interacting with fucoside, fucosyllactose, and plant xyloglucan. J Biol Chem 280: 27839–27849 15923179 10.1074/jbc.M505184200

[koac040-B91] Kubicek CP , Starr TL, Louise Glass N (2014) Plant cell wall–degrading enzymes and their secretion in plant-pathogenic fungi. Annu Rev Phytopathol 52: 427–451 25001456 10.1146/annurev-phyto-102313-045831

[koac040-B92] Kumar V , Hainaut M, Delhomme N, Mannapperuma C, Immerzeel P, Street NR, Henrissat B, Mellerowicz EJ (2019) Poplar carbohydrate-active enzymes: whole-genome annotation and functional analyses based on RNA expression data. Plant J 99: 589–609 31111606 10.1111/tpj.14417 PMC6852159

[koac040-B93] Lai M-W , Liou R-F (2018) Two genes encoding GH10 xylanases are essential for the virulence of the oomycete plant pathogen Phytophthora parasitica. Curr Genet 64: 931–943 29470644 10.1007/s00294-018-0814-z

[koac040-B94] Lamport DTA , Várnai P (2013) Periplasmic arabinogalactan glycoproteins act as a calcium capacitor that regulates plant growth and development. New Phytol 197: 58–64 23106282 10.1111/nph.12005

[koac040-B95] Lee Y , Rubio MC, Alassimone J, Geldner N (2013) A mechanism for localized lignin deposition in the endodermis. Cell 153: 402–412 23541512 10.1016/j.cell.2013.02.045

[koac040-B96] Lee M-H , Jeon HS, Kim SH, Chung JH, Roppolo D, Lee H-J, Cho HJ, Tobimatsu Y, Ralph J, Park OK (2019) Lignin-based barrier restricts pathogens to the infection site and confers resistance in plants. EMBO J 38: e101948 31559647 10.15252/embj.2019101948 PMC6885736

[koac040-B97] Lehmann J , Jørgensen ME, Fratz S, Müller HM, Kusch J, Scherzer S, Navarro-Retamal C, Mayer D, Böhm J, Konrad KR, et al (2021). Acidosis-induced activation of anion channel SLAH3 in the flooding-related stress response of Arabidopsis. Curr Biol 31: 3575–3585.e9 34233161 10.1016/j.cub.2021.06.018

[koac040-B98] Little A , Schwerdt JG, Shirley NJ, Khor SF, Neumann K, O’Donovan LA, Lahnstein J, Collins HM, Henderson M, Fincher GB, et al (2018) Revised phylogeny of the cellulose synthase gene superfamily: insights into cell wall evolution. Plant Physiol 177: 1124–1141 29780036 10.1104/pp.17.01718 PMC6052982

[koac040-B99] Lionetti V , Fabri E, De Caroli M, Hansen AR, Willats WGT, Piro G, Bellincampi D (2017) Three pectin methylesterase inhibitors protect cell wall integrity for arabidopsis immunity to *Botrytis*. Plant Physiol 173: 1844–1863 28082716 10.1104/pp.16.01185 PMC5338656

[koac040-B100] Liu N , Sun Y, Pei Y, Zhang X, Wang P, Li X, Li D, Hou Y (2018) A pectin methylesterase inhibitor enhances resistance to *Verticillium* wilt. Plant Physiol 176: 2202–2220 29363564 10.1104/pp.17.01399 PMC5841709

[koac040-B101] Lombard V , Ramulu HG, Drula E, Coutinho PM, Henrissat B (2014) The carbohydrate-active enzymes database (CAZy) in 2013. Nucleic Acids Res 42: 490–495 10.1093/nar/gkt1178 PMC3965031 24270786

[koac040-B102] López-Coria M , Hernández-Mendoza JL, Sánchez-Nieto S (2016) Trichoderma asperellum induces maize seedling growth by activating the plasma membrane H^+^-ATPase. Mol Plant-Microbe Interact 29: 797–806 27643387 10.1094/MPMI-07-16-0138-R

[koac040-B103] Lopez-Hernandez F , Tryfona T, Rizza A, Yu XL, Harris MOB, Webb AAR, Kotake T, Dupree P (2020) Calcium binding by arabinogalactan polysaccharides is important for normal plant development. Plant Cell 32: 3346–3369 32769130 10.1105/tpc.20.00027 PMC7534474

[koac040-B104] Lu Y , Tsuda K (2021) Intimate association of PRR- and NLR-mediated signaling in plant immunity. Mol Plant Microbe Interact 34: 3–14 33048599 10.1094/MPMI-08-20-0239-IA

[koac040-B105] Mäe A , Heikinheimo R, Palva ET (1995) Structure and regulation of the Erwinia carotovora subspecies carotovora SCC3193 cellulase gene celV1 and the role of cellulase in phytopathogenicity. Mol Gen Genet 247: 17–26 7715600 10.1007/BF00425817

[koac040-B106] Manabe Y , Nafisi M, Verhertbruggen Y, Orfila C, Gille S, Rautengarten C, Cherk C, Marcus SE, Somerville S, Pauly M, et al (2011). Loss-of-function mutation of *REDUCED WALL ACETYLATION2* in Arabidopsis leads to reduced cell wall acetylation and increased resistance to *Botrytis cinerea*. Plant Physiol 155: 1068–1078 21212300 10.1104/pp.110.168989 PMC3046569

[koac040-B107] Masachis S , Segorbe D, Turrà D, Leon-Ruiz M, Fürst U, El Ghalid M, Leonard G, López-Berges MS, Richards TA, Felix G, et al (2016). A fungal pathogen secretes plant alkalinizing peptides to increase infection. Nat Microbiol 1: 16043 27572834 10.1038/nmicrobiol.2016.43

[koac040-B108] Matthysse AG (2014) Attachment of Agrobacterium to plant surfaces. Front Plant Sci 5: 252 24926300 10.3389/fpls.2014.00252 PMC4046570

[koac040-B109] Ma Z , Song T, Zhu L, Ye W, Wang Y, Shao Y, Dong S, Zhang Z, Dou D, Zheng X, et al (2015) A Phytophthora sojae glycoside hydrolase 12 protein is a major virulence factor during soybean infection and is recognized as a PAMP. Plant Cell 27: 2057–2072 26163574 10.1105/tpc.15.00390 PMC4531360

[koac040-B110] McQueen-Mason SJ , Fry SC, Durachko DM, Cosgrove DJ (1993) The relationship between xyloglucan endotransglycosylase and in-vitro cell wall extension in cucumber hypocotyls. Planta 190: 327–331 7763661 10.1007/BF00196961

[koac040-B111] Mecchia MA , Santos-Fernandez G, Duss NN, Somoza SC, Boisson-Dernier A, Gagliardini V, Martínez-Bernardini A, Fabrice TN, Ringli C, Muschietti JP, et al (2017) RALF4/19 peptides interact with LRX proteins to control pollen tube growth in Arabidopsis. Science 358: 1600–1603 29242232 10.1126/science.aao5467

[koac040-B112] Mélida H , Sopeña-Torres S, Bacete L, Garrido-Arandia M, Jordá L, López G, Muñoz-Barrios A, Pacios LF, Molina A (2018) Non-branched β-1,3-glucan oligosaccharides trigger immune responses in Arabidopsis. Plant J 93: 34–49 29083116 10.1111/tpj.13755

[koac040-B113] Mélida H , Bacete L, Ruprecht C, Rebaque D, Del Hierro I, López G, Brunner F, Pfrengle F, Molina A (2020) Arabinoxylan-oligosaccharides act as damage associated molecular patterns in plants regulating disease resistance. Front Plant Sci 11: 1210 32849751 10.3389/fpls.2020.01210 PMC7427311

[koac040-B114] Menna A , Dora S, Sancho-Andrés G, Kashyap A, Meena MK, Sklodowski K, Gasperini D, Coll NS, Sánchez-Rodríguez C (2021a) A primary cell wall cellulose-dependent defense mechanism against vascular pathogens revealed by time-resolved dual transcriptomics. BMC Biol 19: 161 34404410 10.1186/s12915-021-01100-6 PMC8371875

[koac040-B115] Menna A , Dora S, Sancho-Andrés G, Kashyap A, Meena MK, Sklodowski K, Gasperini D, Coll NS, Sánchez-Rodríguez C (2021) A primary cell wall cellulose-dependent defense mechanism against vascular pathogens revealed by time-resolved dual transcriptomics. BMC Biol 19: 161 34404410 10.1186/s12915-021-01100-6 PMC8371875

[koac040-B116] Mishler-Elmore JW , Zhou Y, Sukul A, Oblak M, Tan L, Faik A, Held MA (2021) Extensins: self-assembly, crosslinking, and the role of peroxidases. Front Plant Sci 12: 664738 34054905 10.3389/fpls.2021.664738 PMC8160292

[koac040-B117] Mnich E , Bjarnholt N, Eudes A, Harholt J, Holland C, Jorgensen B, Larsen FH, Liu M, Manat R, Meyer AS, et al (2020) Phenolic cross-links: building and de-constructing the plant cell wall. Nat Prod Rep 37: 919–961 31971193 10.1039/c9np00028c

[koac040-B118] Mohnen D (2008) Pectin structure and biosynthesis. Curr Opin Plant Biol 11: 266–277 18486536 10.1016/j.pbi.2008.03.006

[koac040-B119] Molina A , Miedes E, Bacete L, Rodríguez T, Mélida H, Denancé N, Sánchez-Vallet A, Rivière MP, López G, Freydier A, et al (2021). Cell wall composition determines disease resistance specificity and fitness. Proc Natl Acad Sci USA 118: e2010243118 10.1073/pnas.2010243118 PMC7865177 33509925

[koac040-B120] Moroz N , Fritch KR, Marcec MJ, Tripathi D, Smertenko A, Tanaka K (2017) Extracellular alkalinization as a defense response in potato cells. Front Plant Sci 8: 32 28174578 10.3389/fpls.2017.00032 PMC5258701

[koac040-B121] Morris VJ , Brownsey GJ, Harris JE, Gunning AP, Stevens BJH, Johnston AWB (1989) Cation-dependent gelation of the acidic extracellular polysaccharides of Rhizobium leguminosarum: a non-specific mechanism for the attachment of bacteria to plant roots. Carbohydr Res 191: 315–320

[koac040-B122] Müller N , Leroch M, Schumacher J, Zimmer D, Könnel A, Klug K, Leisen T, Scheuring D, Sommer F, Mühlhaus T, et al (2018) Investigations on VELVET regulatory mutants confirm the role of host tissue acidification and secretion of proteins in the pathogenesis of Botrytis cinerea. New Phytol 219: 1062–1074 29790574 10.1111/nph.15221

[koac040-B123] Narváez-Barragán DA , Tovar-Herrera OE, Segovia L, Serrano M, Martinez-Anaya C (2020) Expansin-related proteins: biology, microbe-plant interactions and associated plant-defense responses. Microbiology 166: 1007–1018 33141007 10.1099/mic.0.000984

[koac040-B124] Navarrete F , Grujic N, Stirnberg A, Saado I, Aleksza D, Gallei M, Adi H, Alcântara A, Khan M, Bindics J, et al (2021). The Pleiades are a cluster of fungal effectors that inhibit host defenses. PLoS Pathog 17: e1009641 34166468 10.1371/journal.ppat.1009641 PMC8224859

[koac040-B125] Nothnagel EA , McNeil M, Albersheim P, Dell A (1983) Host-pathogen interactions: XXII. A galacturonic acid oligosaccharide from plant cell walls elicits phytoalexins. Plant Physiol 71: 916–926 16662929 10.1104/pp.71.4.916 PMC1066144

[koac040-B126] Novaković L , Guo T, Bacic A, Sampathkumar A, Johnson KL (2018) Hitting the wall-sensing and signaling pathways involved in plant cell wall remodeling in response to abiotic stress. Plants 7: 89 10.3390/plants7040089 PMC6313904 30360552

[koac040-B127] Novo M , Silvar C, Merino F, Martínez-Cortés T, Lu F, Ralph J, Pomar F (2017) Deciphering the role of the phenylpropanoid metabolism in the tolerance of Capsicum annuum L. to Verticillium dahliae Kleb. Plant Sci 258: 12–20 28330555 10.1016/j.plantsci.2017.01.014

[koac040-B128] Nühse TS , Bottrill AR, Jones AME, Peck SC (2007) Quantitative phosphoproteomic analysis of plasma membrane proteins reveals regulatory mechanisms of plant innate immune responses. Plant J 51: 931–940 17651370 10.1111/j.1365-313X.2007.03192.x PMC2156193

[koac040-B129] Nunes CS , Kunamneni A (2018) Laccases—properties and applications. *In* Nunes CS, Kumar V, eds, Enzymes in Human and Animal Nutrition. Academic Press, pp. 133–161. https://doi.org/10.1016/B978-0-12-805419-2.00001-0

[koac040-B130] Oide S , Tanaka Y, Watanabe A, Inui M (2019) Carbohydrate-binding property of a cell wall integrity and stress response component (WSC) domain of an alcohol oxidase from the rice blast pathogen Pyricularia oryzae. Enzyme Microb Technol 125: 13–20 30885320 10.1016/j.enzmictec.2019.02.009

[koac040-B132] Paccanaro MC , Sella L, Castiglioni C, Giacomello F, Martínez-Rocha AL, D’Ovidio R, Schäfer W, Favaron F (2017) Synergistic effect of different plant cell wall–degrading enzymes is important for virulence of Fusarium graminearum. Mol Plant-Microbe Interact 30: 886–895 28800710 10.1094/MPMI-07-17-0179-R

[koac040-B133] Paredez AR , Somerville CR, Ehrhardt DW (2006) Visualization of cellulose synthase demonstrates functional association with microtubules. Science 312: 1491–1495 16627697 10.1126/science.1126551

[koac040-B134] Park YB , Cosgrove DJ (2012a) A revised architecture of primary cell walls based on biomechanical changes induced by substrate-specific endoglucanases. Plant Physiol 158: 1933–1943 22362871 10.1104/pp.111.192880 PMC3320196

[koac040-B135] Park YB , Cosgrove DJ (2012b) Changes in cell wall biomechanical properties in the xyloglucan-deficient xxt1/xxt2 mutant of Arabidopsis. Plant Physiol 158: 465–475 22108526 10.1104/pp.111.189779 PMC3252101

[koac040-B136] Pauly M , Ramírez V (2018) New insights into wall polysaccharide O-acetylation. Front Plant Sci 9, 10.3389/fpls.2018.01210 PMC6110886 30186297

[koac040-B137] Pedreño MA , Ros Barceló A, Sabater F, Muñoz R (1989) Control by pH of cell wall peroxidase activity involved in lignification. Plant Cell Physiol 30: 237–241 10.1111/j.1399-3054.1990.tb00033.x 21087268

[koac040-B138] Pelagio-Flores R , Esparza-Reynoso S, Garnica-Vergara A, López-Bucio J, Herrera-Estrella A (2017) Trichoderma-induced acidification is an early trigger for changes in Arabidopsis root growth and determines fungal phytostimulation. Front Plant Sci 8: 822 28567051 10.3389/fpls.2017.00822 PMC5434454

[koac040-B139] Pellerin P , Doco T, Vidal S, Williams P, Brillouet J, O’Neill M (1996) Structural characterization of red wine rhamnogalacturonan II. Carbohydr Res 290: 183–197 8823907 10.1016/0008-6215(96)00139-5

[koac040-B140] Philippe G , Sørensen I, Jiao C, Sun X, Fei Z, Domozych DS, Rose JK (2020) Cutin and suberin: assembly and origins of specialized lipidic cell wall scaffolds. Curr Opin Plant Biol 55: 11–20 32203682 10.1016/j.pbi.2020.01.008

[koac040-B141] Phyo P , Gu Y, Hong M (2019) Impact of acidic pH on plant cell wall polysaccharide structure and dynamics: insights into the mechanism of acid growth in plants from solid-state NMR. Cellulose 26: 291–304

[koac040-B142] Phyo P , Wang T, Kiemle SN, O’Neill H, Pingali SV, Hong M, Cosgrove DJ (2017a) Gradients in wall mechanics and polysaccharides along growing inflorescence stems. Plant Physiol 175: 1593–1607 29084904 10.1104/pp.17.01270 PMC5717741

[koac040-B143] Phyo P , Wang T, Xiao C, Anderson CT, Hong M (2017b) Effects of pectin molecular weight changes on the structure, dynamics, and polysaccharide interactions of primary cell walls of Arabidopsis thaliana: insights from solid-state NMR. Biomacromolecules 18: 2937–2950 28783321 10.1021/acs.biomac.7b00888

[koac040-B144] Pimentel D , Amaro R, Erban A, Mauri N, Soares F, Rego C, Martínez-Zapater JM, Mithöfer A, Kopka J, Fortes AM (2021) Transcriptional, hormonal, and metabolic changes in susceptible grape berries under powdery mildew infection. J Exp Bot 72: 6544–6569 34106234 10.1093/jxb/erab258

[koac040-B145] Pontiggia D , Benedetti M, Costantini S, De Lorenzo G, Cervone F (2020) Dampening the DAMPs: how plants maintain the homeostasis of cell wall molecular patterns and avoid hyper-immunity. Front Plant Sci 11: 613259 33391327 10.3389/fpls.2020.613259 PMC7773757

[koac040-B146] Pouzoulet J , Scudiero E, Schiavon M, Rolshausen PE (2017) Xylem vessel diameter affects the compartmentalization of the vascular pathogen Phaeomoniella chlamydospora in grapevine. Front Plant Sci 8: 1442 28871268 10.3389/fpls.2017.01442 PMC5566965

[koac040-B147] Plieth C , Vollbehr S (2012) Calcium promotes activity and confers heat stability on plant peroxidases. Plant Signal Behav 7: 650–660 22580695 10.4161/psb.20065 PMC3442860

[koac040-B148] Preis I , Abramson M, Shoseyov O (2018) The modification of cell wall properties by expression of recombinant resilin in transgenic plants. Mol Biotechnol 60: 310–318 29511941 10.1007/s12033-018-0074-7

[koac040-B149] Prusky D , McEvoy JL, Leverentz B, Conway WS (2001) Local modulation of host pH by Colletotrichum species as a mechanism to increase virulence. Mol Plant Microbe Interact 14: 1105–1113 11551075 10.1094/MPMI.2001.14.9.1105

[koac040-B151] Rajarammohan S (2021) Redefining plant-necrotroph interactions: the thin line between hemibiotrophs and necrotrophs. Front Microbiol 12: 673518 33995337 10.3389/fmicb.2021.673518 PMC8113614

[koac040-B152] Rajeshwari R , Jha G, Sonti RV (2005) Role of an in planta-expressed xylanase of Xanthomonas oryzae pv. oryzae in promoting virulence on rice. Mol Plant Microbe Interact 18: 830–837 16134895 10.1094/MPMI-18-0830

[koac040-B153] Ralph J , Lundquist K, Brunow G, Lu F, Kim H, Schatz PF, Marita JM, Hatfield RD, Ralph SA, Christensen JH, et al (2004) Lignins: natural polymers from oxidative coupling of 4-hydroxyphenyl-propanoids. Phytochem Rev 3: 29–60

[koac040-B154] Ramírez V , Pauly M (2019) Genetic dissection of cell wall defects and the strigolactone pathway in Arabidopsis. Plant Direct 3: 1–11 10.1002/pld3.149 PMC6589044 31245785

[koac040-B155] Ranathunge K , Thomas RH, Fang X, Peterson CA, Gijzen M, Bernards MA (2008) Soybean root suberin and partial resistance to root rot caused by Phytophthora sojae. Phytopathology 98: 1179–1189 18943406 10.1094/PHYTO-98-11-1179

[koac040-B156] Ranty B , Aldon D, Cotelle V, Galaud J-P, Thuleau P, Mazars C (2016) Calcium sensors as key hubs in plant responses to biotic and abiotic stresses. Front Plant Sci 7: 327 27014336 10.3389/fpls.2016.00327 PMC4792864

[koac040-B157] Rebaque D , Del Hierro I, López G, Bacete L, Vilaplana F, Dallabernardina P, Pfrengle F, Jordá L, Sánchez-Vallet A, Pérez R, et al (2021) Cell wall-derived mixed-linked β-1,3/1,4-glucans trigger immune responses and disease resistance in plants. Plant J 106: 601–615 33544927 10.1111/tpj.15185 PMC8252745

[koac040-B158] Renard CMGC , Renard CMG, Jarvis MC (1999) Acetylation and methylation of homogalacturonans 2: effect on ion-binding properties and conformations. Carbohydr Polym 39: 209–216

[koac040-B159] Rich MK , Schorderet M, Reinhardt D (2014) The role of the cell wall compartment in mutualistic symbioses of plants. Front Plant Sci 5: 238 24917869 10.3389/fpls.2014.00238 PMC4041022

[koac040-B160] Robb J , Lee SW, Mohan R, Kolattukudy PE (1991) Chemical characterization of stress-induced vascular coating in tomato. Plant Physiol 97: 528–536 16668431 10.1104/pp.97.2.528 PMC1081039

[koac040-B161] Rojas-Murcia N , Hématy K, Lee Y, Emonet A, Ursache R, Fujita S, De Bellis D, Geldner N (2020) High-order mutants reveal an essential requirement for peroxidases but not laccases in Casparian strip lignification. Proc Natl Acad Sci USA 117: 29166–29177 33139576 10.1073/pnas.2012728117 PMC7682338

[koac040-B162] Sabbadin F , Urresti S, Henrissat B, Avrova AO, Welsh LRJ, Lindley PJ, Csukai M, Squires JN, Walton PH, Davies GJ, et al (2021). Secreted pectin monooxygenases drive plant infection by pathogenic oomycetes. Science 373: 774–779 34385392 10.1126/science.abj1342

[koac040-B163] Sabella E , Luvisi A, Aprile A, Negro C, Vergine M, Nicolì F, Miceli A, De Bellis L (2018) Xylella fastidiosa induces differential expression of lignification related-genes and lignin accumulation in tolerant olive trees cv. Leccino. J Plant Physiol 220: 60–68 29149645 10.1016/j.jplph.2017.10.007

[koac040-B164] Saijo Y , Loo EP-I, Yasuda S (2018) Pattern recognition receptors and signaling in plant-microbe interactions. Plant J 93: 592–613 29266555 10.1111/tpj.13808

[koac040-B165] Sampedro J , Cosgrove DJ (2005) The expansin superfamily. Genome Biol 6: 242 16356276 10.1186/gb-2005-6-12-242 PMC1414085

[koac040-B166] Sattelmacher B (2001) The apoplast and its significance for plant mineral nutrition. New Phytol 149: 167–192 33874640 10.1046/j.1469-8137.2001.00034.x

[koac040-B167] Scheller HV , Ulvskov P (2010) Hemicelluloses. Annu Rev Plant Biol 61: 263–289 20192742 10.1146/annurev-arplant-042809-112315

[koac040-B168] Schultz CJ , Rumsewicz MP, Johnson KL, Jones BJ, Gaspar YM, Bacic A (2002) Using genomic resources to guide research directions. The arabinogalactan protein gene family as a test case. Plant Physiol 129: 1448–1463 12177459 10.1104/pp.003459 PMC166734

[koac040-B169] Sella L , Castiglioni C, Paccanaro MC, Janni M, Schäfer W, D’Ovidio R, Favaron F (2016) Involvement of fungal pectin methylesterase activity in the interaction between Fusarium graminearum and wheat. Mol Plant Microbe Interact 29: 258–267 26713352 10.1094/MPMI-07-15-0174-R

[koac040-B170] Sénéchal F , Habrylo O, Hocq L, Domon J-M, Marcelo P, Lefebvre V, Pelloux J, Mercadante D (2017) Structural and dynamical characterization of the pH-dependence of the pectin methylesterase-pectin methylesterase inhibitor complex. J Biol Chem 292: 21538–21547 29109147 10.1074/jbc.RA117.000197 PMC5766959

[koac040-B171] Shah K , Penel C, Gagnon J, Dunand C (2004) Purification and identification of a Ca(2+)-pectate binding peroxidase from Arabidopsis leaves. Phytochemistry 65: 307–312 14751301 10.1016/j.phytochem.2003.11.019

[koac040-B172] Sharma P , Jha AB, Dubey RS, Pessarakli M (2012) Reactive oxygen species, oxidative damage, and antioxidative defense mechanism in plants under stressful conditions. J Bot 2012. doi:10.1155/2012/217037

[koac040-B173] Sharmin S , Azam MS, Islam MS, Sajib AA, Mahmood N, Hasan AMM, Ahmed R, Sultana K, Khan H (2012) Xyloglucan endotransglycosylase/hydrolase genes from a susceptible and resistant jute species show opposite expression pattern following *Macrophomina phaseolina* infection. Commun Integr Biol 5: 598–606 23336031 10.4161/cib.21422 PMC3541328

[koac040-B174] Shi YZ , Zhu XF, Miller JG, Gregson T, Zheng SJ, Fry SC (2015) Distinct catalytic capacities of two aluminium-repressed Arabidopsis thaliana xyloglucan endotransglucosylase/hydrolases, XTH15 and XTH31, heterologously produced in Pichia. Phytochemistry 112: 160–169 25446234 10.1016/j.phytochem.2014.09.020

[koac040-B175] Shukla V , Han J-P, Cléard F, Legendre LL, Gully K, Flis P, Berhin A, Andersen TG, Salt DE, Nawrath C, et al (2021). Suberin plasticity to developmental and exogenous cues is regulated by a set of MYB transcription factors. Proc Natl Acad Sci USA 118: e2101730118 34551972 10.1073/pnas.2101730118 PMC8488582

[koac040-B176] Simmons TJ , Mortimer JC, Bernardinelli OD, Pöppler A-C, Brown SP, deAzevedo ER, Dupree R, Dupree P (2016) Folding of xylan onto cellulose fibrils in plant cell walls revealed by solid-state NMR. Nat Commun 7: 13902 28000667 10.1038/ncomms13902 PMC5187587

[koac040-B177] Singh D , Dutta TK, Shivakumara TN, Dash M, Bollinedi H, Rao U (2021) Suberin biopolymer in rice root exodermis reinforces preformed barrier against Meloidogyne graminicola infection. Rice Sci 28: 301–312

[koac040-B178] Smit G , Swart S, Lugtenberg BJ, Kijne JW (1992) Molecular mechanisms of attachment of Rhizobium bacteria to plant roots. Mol Microbiol 6: 2897–2903 1479881 10.1111/j.1365-2958.1992.tb01748.x

[koac040-B179] Somerville C (2006) Cellulose synthesis in higher plants. Annu Rev Cell Dev Biol 22: 53–78 16824006 10.1146/annurev.cellbio.22.022206.160206

[koac040-B180] Survila M , Davidsson PR, Pennanen V, Kariola T, Broberg M, Sipari N, Heino P, Palva ET (2016) Peroxidase-generated apoplastic ROS impair cuticle integrity and contribute to DAMP-elicited defenses. Front Plant Sci 7: 1945 28066496 10.3389/fpls.2016.01945 PMC5179520

[koac040-B181] Swaminathan S , Reem NT, Lionetti V, Zabotina OA (2021) Coexpression of fungal cell wall-modifying enzymes reveals their additive impact on Arabidopsis resistance to the fungal pathogen, *Botrytis cinerea*. Biology 10: 1070 34681168 10.3390/biology10101070 PMC8533531

[koac040-B182] Tan L , Eberhard S, Pattathil S, Warder C, Glushka J, Yuan C, Hao Z, Zhu X, Avci U, Miller JS, et al (2013) An *Arabidopsis* cell wall proteoglycan consists of pectin and arabinoxylan covalently linked to an arabinogalactan protein. Plant Cell 25: 270–287 23371948 10.1105/tpc.112.107334 PMC3584541

[koac040-B183] Tan L , Tees D, Qian J, Kareem S, Kieliszewski MJ (2018) Intermolecular interactions between glycomodules of plant cell wall arabinogalactan-proteins and extensins. Cell Surf 1: 25–33 32743125 10.1016/j.tcsw.2018.03.001 PMC7389152

[koac040-B184] Temple H , Phyo P, Yang W, Lyczakowski JJ, Echevarría-Poza A, Yakunin I, Parra-Rojas JP, Terrett MM, Saez-Aguayo S, Dupree R, et al (2021). Discovery of putative Golgi S-Adenosyl methionine transporters reveals the importance of plant cell wall polysaccharide methylation. doi: org/10.1101/2021.07.06.451061 10.1038/s41477-022-01156-4 35681018

[koac040-B185] Terrett OM , Dupree P (2019) Covalent interactions between lignin and hemicelluloses in plant secondary cell walls. Curr Opin Biotechnol 56: 97–104 30423528 10.1016/j.copbio.2018.10.010

[koac040-B186] Terrett OM , Lyczakowski JJ, Yu L, Iuga D, Franks WT, Brown SP, Dupree R, Dupree P (2019) Molecular architecture of softwood revealed by solid-state NMR. Nat Commun 10: 4978 31673042 10.1038/s41467-019-12979-9 PMC6823442

[koac040-B187] Thilini Chethana KW , Peng J, Li X, Xing Q, Liu M, Zhang W, Hyde KD, Zhao W, Yan J (2020) LtEPG1, a secretory endopolygalacturonase protein, regulates the virulence of in and is recognized as a microbe-associated molecular patterns. Phytopathology 110: 1727–1736 32460690 10.1094/PHYTO-04-20-0118-R

[koac040-B188] Thor K , Jiang S, Michard E, George J, Scherzer S, Huang S, Dindas J, Derbyshire P, Leitão N, DeFalco TA, et al (2020) The calcium-permeable channel OSCA1.3 regulates plant stomatal immunity. Nature 585: 569–573 32846426 10.1038/s41586-020-2702-1 PMC8435934

[koac040-B189] Thor K , Peiter E (2014) Cytosolic calcium signals elicited by the pathogen-associated molecular pattern flg22 in stomatal guard cells are of an oscillatory nature. New Phytol 204: 873–881 25243759 10.1111/nph.13064

[koac040-B190] Tian W , Hou C, Ren Z, Wang C, Zhao F, Dahlbeck D, Hu S, Zhang L, Niu Q, Li L, et al (2019). A calmodulin-gated calcium channel links pathogen patterns to plant immunity. Nature 572: 131–135 31316205 10.1038/s41586-019-1413-y

[koac040-B191] Tobimatsu Y , Schuetz M (2019) Lignin polymerization: how do plants manage the chemistry so well? Curr Opin Biotechnol 56: 75–81 30359808 10.1016/j.copbio.2018.10.001

[koac040-B192] Torres MA (2010) ROS in biotic interactions. Physiol Plant 138: 414–429 20002601 10.1111/j.1399-3054.2009.01326.x

[koac040-B193] Tryfona T , Liang H-C, Kotake T, Tsumuraya Y, Stephens E, Dupree P (2012) Structural characterization of Arabidopsis leaf arabinogalactan polysaccharides. Plant Physiol 160: 653–666 22891237 10.1104/pp.112.202309 PMC3461546

[koac040-B194] Valiante V (2017) The cell wall integrity signaling pathway and its involvement in secondary metabolite production. J Fungi (Basel) 3: 68 10.3390/jof3040068 PMC5753170 29371582

[koac040-B195] Van Sandt VST , Suslov D, Verbelen J-P, Vissenberg K (2007) Xyloglucan endotransglucosylase activity loosens a plant cell wall. Ann Bot 100: 1467–1473 17916584 10.1093/aob/mcm248 PMC2759230

[koac040-B196] Van Vu B , Itoh K, Nguyen QB, Tosa Y, Nakayashiki H (2012) Cellulases belonging to glycoside hydrolase families 6 and 7 contribute to the virulence of Magnaporthe oryzae. Mol Plant Microbe Interact 25: 1135–1141 22852807 10.1094/MPMI-02-12-0043-R

[koac040-B197] Vera A , Moreno JL, Siles JA, López-Mondejar R, Zhou Y, Li Y, García C, Nicolás E, Bastida F (2021). Interactive impacts of boron and organic amendments in plant-soil microbial relationships. J Hazard Mater 408: 124939 33383449 10.1016/j.jhazmat.2020.124939

[koac040-B198] Verna J , Lodder A, Lee K, Vagts A, Ballester R (1997) A family of genes required for maintenance of cell wall integrity and for the stress response in Saccharomyces cerevisiae. Proc Natl Acad Sci USA 94: 13804–13809 9391108 10.1073/pnas.94.25.13804 PMC28388

[koac040-B199] Villa-Rivera MG , Cano-Camacho H, López-Romero E, Zavala-Páramo MG (2021) The role of arabinogalactan type II degradation in plant-microbe interactions. Front Microbiol 12: 730543 34512607 10.3389/fmicb.2021.730543 PMC8424115

[koac040-B200] Vishwanath SJ , Delude C, Domergue F, Rowland O (2015) Suberin: biosynthesis, regulation, and polymer assembly of a protective extracellular barrier. Plant Cell Rep 34: 573–586 25504271 10.1007/s00299-014-1727-z

[koac040-B201] Vrsanská M , Kolenová K, Puchart V, Biely P (2007) Mode of action of glycoside hydrolase family 5 glucuronoxylan xylanohydrolase from Erwinia chrysanthemi. FEBS J 274: 1666–1677 17381510 10.1111/j.1742-4658.2007.05710.x

[koac040-B202] Wang D , Chen J-Y, Song J, Li J-J, Klosterman SJ, Li R, Kong Z-Q, Subbarao KV, Dai X-F, Zhang D-D (2021a) Cytotoxic function of xylanase VdXyn4 in the plant vascular wilt pathogen Verticillium dahliae. Plant Physiol 187: 409–429 34618145 10.1093/plphys/kiab274 PMC8418393

[koac040-B203] Wang S , Vetukuri RR, Kushwaha SK, Hedley PE, Morris J, Studholme DJ, Welsh LRJ, Boevink PC, Birch PRJ, Whisson SC (2021b) Haustorium formation and a distinct biotrophic transcriptome characterize infection of Nicotiana benthamiana by the tree pathogen Phytophthora kernoviae. Mol Plant Pathol 22: 954–968 34018655 10.1111/mpp.13072 PMC8295517

[koac040-B204] Wang T , Chen Y, Tabuchi A, Cosgrove DJ, Hong M (2016) The target of β-expansin EXPB1 in maize cell walls from binding and solid-state NMR studies. Plant Physiol 172: 2107–2119 27729469 10.1104/pp.16.01311 PMC5129719

[koac040-B205] Wang T , Park YB, Caporini MA, Rosay M, Zhong L, Cosgrove DJ, Hong M (2013) Sensitivity-enhanced solid-state NMR detection of expansin’s target in plant cell walls. Proc Natl Acad Sci USA 110: 16444–16449 24065828 10.1073/pnas.1316290110 PMC3799313

[koac040-B206] Wang T , Park YB, Cosgrove DJ, Hong M (2015) Cellulose-pectin spatial contacts are inherent to never-dried Arabidopsis primary cell walls: evidence from solid-state nuclear magnetic resonance. Plant Physiol 168: 871–884 26036615 10.1104/pp.15.00665 PMC4741345

[koac040-B207] Wang X , Wilson L, Cosgrove DJ (2020a) Pectin methylesterase selectively softens the onion epidermal wall yet reduces acid-induced creep. J Exp Bot 71: 2629–2640 32006044 10.1093/jxb/eraa059 PMC7210771

[koac040-B208] Wang Y , Li X, Fan B, Zhu C, Chen Z (2021c) Regulation and function of defense-related callose deposition in plants. Int J Mol Sci 22: 2393 10.3390/ijms22052393 PMC7957820 33673633

[koac040-B209] Wang Y , Li Y, Li S, Li Q, Fan W, Kiatoukosin L, Chen J (2019) Extracellular polysaccharides of endophytic fungus Alternaria tenuissima F1 from Angelica sinensis: production conditions, purification, and antioxidant properties. Int J Biol Macromol 133: 172–183 30951779 10.1016/j.ijbiomac.2019.03.246

[koac040-B210] Wang Y , Wang Y, Wang Y (2020b) Apoplastic proteases: powerful weapons against pathogen infection in plants. Plant Commun 1: 100085 33367249 10.1016/j.xplc.2020.100085 PMC7748006

[koac040-B211] Wanke A , Rovenich H, Schwanke F, Velte S, Becker S, Hehemann J-H, Wawra S, Zuccaro A (2020) Plant species-specific recognition of long and short β-1,3-linked glucans is mediated by different receptor systems. Plant J 102: 1142–1156 31925978 10.1111/tpj.14688

[koac040-B212] Westphal L , Strehmel N, Eschen-Lippold L, Bauer N, Westermann B, Rosahl S, Scheel D, Lee J (2019) pH effects on plant calcium fluxes: lessons from acidification-mediated calcium elevation induced by the γ-glutamyl-leucine dipeptide identified from Phytophthora infestans. Sci Rep 9: 4733 30894659 10.1038/s41598-019-41276-0 PMC6426842

[koac040-B213] Williams A , Wilkinson A, Krehenbrink M, Russo DM, Zorreguieta A, Downie JA (2008) Glucomannan-mediated attachment of Rhizobium leguminosarum to pea root hairs is required for competitive nodule infection. J Bacteriol 190: 4706–4715 18441060 10.1128/JB.01694-07 PMC2446804

[koac040-B214] Wimmer MA , Eichert T (2013) Review: mechanisms for boron deficiency-mediated changes in plant water relations. Plant Sci 203–204: 25–32 10.1016/j.plantsci.2012.12.012 23415325

[koac040-B215] Wood AKM , Walker C, Lee W-S, Urban M, Hammond-Kosack KE (2020) Functional evaluation of a homologue of plant rapid alkalinisation factor (RALF) peptides in Fusarium graminearum. Fungal Biol 124: 753–765 32883427 10.1016/j.funbio.2020.05.001 PMC7487784

[koac040-B216] Wormit A , Usadel B (2018) The multifaceted role of pectin methylesterase inhibitors (PMEIs). Int J Mol Sci 19: 2878 10.3390/ijms19102878 PMC6213510 30248977

[koac040-B217] Wu J , Wang Y, Park S-Y, Kim SG, Yoo JS, Park S, Gupta R, Kang KY, Kim ST (2016) Secreted alpha-N-arabinofuranosidase B protein is required for the full virulence of Magnaporthe oryzae and triggers host defences. PLoS One 11: e0165149 27764242 10.1371/journal.pone.0165149 PMC5072668

[koac040-B218] Wu H-C , Bulgakov VP, Jinn T-L (2018) Pectin methylesterases: cell wall remodeling proteins are required for plant response to heat stress. Front Plant Sci 9, https://doi.org/10.3389/fpls.2018.01612 10.3389/fpls.2018.01612 PMC6232315 30459794

[koac040-B219] Xiong X-P , Sun S-C, Zhu Q-H, Zhang X-Y, Liu F, Li Y-J, Xue F, Sun J (2021) Transcriptome analysis and RNA interference reveal GhGDH2 regulating cotton resistance to verticillium wilt by JA and SA signaling pathways. Front Plant Sci 12: 654676 34177978 10.3389/fpls.2021.654676 PMC8226099

[koac040-B220] Xue R , Feng M, Chen J, Ge W, Blair MW (2021) A methyl esterase 1 (PvMES1) promotes the salicylic acid pathway and enhances Fusarium wilt resistance in common beans. Theor Appl Genet 134: 2379–2398 34128089 10.1007/s00122-021-03830-1

[koac040-B221] Yang C , Liu R, Pang J, Ren B, Zhou H, Wang G, Wang E, Liu J (2021) Poaceae-specific cell wall-derived oligosaccharides activate plant immunity via OsCERK1 during Magnaporthe oryzae infection in rice. Nat Commun 12: 2178 33846336 10.1038/s41467-021-22456-x PMC8042013

[koac040-B222] Yang Y , Zhang Y, Li B, Yang X, Dong Y, Qiu D (2018) A pectate lyase induces plant immune responses and contributes to virulence. Front Plant Sci 9: 1271 30271415 10.3389/fpls.2018.01271 PMC6146025

[koac040-B223] Yip Delormel T , Boudsocq M (2019) Properties and functions of calcium-dependent protein kinases and their relatives in Arabidopsis thaliana. New Phytol 224: 585–604 31369160 10.1111/nph.16088

[koac040-B224] York WS , Darvill AG, Albersheim P (1984) Inhibition of 2,4-dichlorophenoxyacetic acid-stimulated elongation of pea stem segments by a xyloglucan oligosaccharide. Plant Physiol 75: 295–297 16663614 10.1104/pp.75.2.295 PMC1066900

[koac040-B225] Yu K , Liu Y, Tichelaar R, Savant N, Lagendijk E, van Kuijk SJL, Stringlis IA, van Dijken AJH, Pieterse CMJ, Bakker PAHM, et al (2019). Rhizosphere-associated pseudomonas suppress local root immune responses by gluconic acid-mediated lowering of environmental pH. Curr Biol 29: 3913–3920.e4 31668625 10.1016/j.cub.2019.09.015

[koac040-B226] Yun MH , Torres PS, El Oirdi M, Rigano LA, Gonzalez-Lamothe R, Marano MR, Castagnaro AP, Dankert MA, Bouarab K, Vojnov AA (2006) Xanthan induces plant susceptibility by suppressing callose deposition. Plant Physiol 141: 178–187 16531487 10.1104/pp.105.074542 PMC1459321

[koac040-B227] Zarattini M , Corso M, Kadowaki MA, Monclaro A, Magri S, Milanese I, Jolivet S, Ortiz de Godoy M, Hermans C, Fagard M, et al (2021) LPMO-oxidized cellulose oligosaccharides evoke immunity in Arabidopsis conferring resistance towards necrotrophic fungus *B. cinerea*. Commun Biol 4: 727 34117349 10.1038/s42003-021-02226-7 PMC8196058

[koac040-B228] Zdunek A , Pieczywek PM, Cybulska J (2021) The primary, secondary, and structures of higher levels of pectin polysaccharides. Compr Rev Food Sci Food Saf 20: 1101–1117 33331080 10.1111/1541-4337.12689

[koac040-B229] Zhang L , Hua C, Pruitt RN, Qin S, Wang L, Albert I, Albert M, van Kan JAL, Nürnberger T (2021a) Distinct immune sensor systems for fungal endopolygalacturonases in closely related Brassicaceae. Nat Plants 7: 1254–1263 34326531 10.1038/s41477-021-00982-2

[koac040-B230] Zhang L , Yan J, Fu Z, Shi W, Ninkuu V, Li G, Yang X, Zeng H (2021b) FoEG1, a secreted glycoside hydrolase family 12 protein from Fusarium oxysporum, triggers cell death and modulates plant immunity. Mol Plant Pathol 22: 522–538 33675158 10.1111/mpp.13041 PMC8035634

[koac040-B231] Zhang T , Vavylonis D, Durachko DM, Cosgrove DJ (2017) Nanoscale movements of cellulose microfibrils in primary cell walls. Nat Plants 3: 17056 28452988 10.1038/nplants.2017.56 PMC5478883

[koac040-B232] Zhang Y , Wu L, Wang X, Chen B, Zhao J, Cui J, Li Z, Yang J, Wu L, Wu J, et al (2019). The cotton laccase gene GhLAC15 enhances Verticillium wilt resistance via an increase in defence-induced lignification and lignin components in the cell walls of plants. Mol Plant Pathol 20: 309–322 30267563 10.1111/mpp.12755 PMC6637971

[koac040-B233] Zhang Y , Yu J, Wang X, Durachko DM, Zhang S, Cosgrove DJ (2021c) Molecular insights into the complex mechanics of plant epidermal cell walls. Science 372: 706–711 33986175 10.1126/science.abf2824

[koac040-B234] Zhao Z , Liu H, Wang C, Xu J-R (2013) Comparative analysis of fungal genomes reveals different plant cell wall degrading capacity in fungi. BMC Genomics 14: 274 23617724 10.1186/1471-2164-14-274 PMC3652786

[koac040-B235] Zhou J-M , Zhang Y (2020) Plant immunity: danger perception and signaling. Cell 181: 978–989 32442407 10.1016/j.cell.2020.04.028

[koac040-B236] Zhu W , Ronen M, Gur Y, Minz-Dub A, Masrati G, Ben-Tal N, Savidor A, Sharon I, Eizner E, Valerius O, et al (2017) BcXYG1, a secreted xyloglucanase from *botrytis cinerea*, triggers both cell death and plant immune responses. Plant Physiol 175: 438–456 28710128 10.1104/pp.17.00375 PMC5580746

[koac040-B237] Zipfel C , Oldroyd GED (2017) Plant signalling in symbiosis and immunity. Nature 543: 328–336 28300100 10.1038/nature22009

